# Envisioning a resilient future for biodiversity conservation in the wake of the COVID‐19 pandemic

**DOI:** 10.1002/pan3.10262

**Published:** 2021-09-28

**Authors:** Ruth H. Thurstan, Kimberley J. Hockings, Johanna S. U. Hedlund, Elena Bersacola, Claire Collins, Regan Early, Yunsiska Ermiasi, Frauke Fleischer‐Dogley, Gabriella Gilkes, Mark E. Harrison, Muhammad Ali Imron, Christopher N. Kaiser‐Bunbury, Daniel Refly Katoppo, Cheryl Marriott, Marie‐May Muzungaile, Ana Nuno, Aissa Regalla de Barros, Frank van Veen, Isuru Wijesundara, Didier Dogley, Nancy Bunbury

**Affiliations:** ^1^ Centre for Ecology and Conservation College of Life and Environmental Sciences University of Exeter Penryn UK; ^2^ Department of Biology Lund University Lund Sweden; ^3^ Institute of Zoology Zoological Society of London London UK; ^4^ Yayasan Borneo Nature Indonesia Central Kalimantan Palangka Raya Indonesia; ^5^ Seychelles Islands Foundation Victoria Republic of Seychelles; ^6^ Eden Project International Ltd Cornwall UK; ^7^ Borneo Nature Foundation International Tremough Innovation Centre Penryn UK; ^8^ School of Geography, Geology and the Environment University of Leicester Leicester UK; ^9^ Faculty of Forestry Universitas Gadjah Mada Yogyakarta Indonesia; ^10^ Cornwall Wildlife Trust Allet UK; ^11^ Biodiversity Conservation and Management Division Ministry of Environment, Energy and Climate Change Victoria Republic of Seychelles; ^12^ Interdisciplinary Centre of Social Sciences (CICS.NOVA) School of Social Sciences and Humanities (NOVA FCSH) NOVA University Lisbon Lisboa Portugal; ^13^ Instituto da Biodiversidade e das Áreas Protegidas Dr. Alfredo Simão da Silva (IBAP) Bissau Guiné‐Bissau; ^14^ Oceanswell Colombo 5 Sri Lanka; ^15^ Inspire for Tomorrow Consultancy Mahé Seychelles

**Keywords:** coronavirus, human–wildlife interactions, SARS‐CoV‐2, shocks, social–ecological systems, sustainability, tourism, zoonotic transmission

## Abstract

1. As the COVID‐19 pandemic continues to affect societies across the world, the ongoing economic and social disruptions are likely to present fundamental challenges for current and future biodiversity conservation.
2. We review the literature for outcomes of past major societal, political, economic and zoonotic perturbations on biodiversity conservation, and demonstrate the complex implications of perturbation events upon conservation efforts. Building on the review findings, we use six in‐depth case studies and the emerging literature to identify positive and negative outcomes of the COVID‐19 pandemic, known and anticipated, for biodiversity conservation efforts around the world.
3. A number of similarities exist between the current pandemic and past perturbations, with experiences highlighting that the pandemic‐induced declines in conservation revenue and capacity, livelihood and trade disruptions are likely to have long‐lasting and negative implications for biodiversity and conservation efforts.
4. Yet, the COVID‐19 pandemic also brought about a global pause in human movement that is unique in recent history, and may yet foster long‐lasting behavioural and societal changes, presenting opportunities to strengthen and advance conservation efforts in the wake of the pandemic. Enhanced collaborations and partnerships at the local level, cross‐sectoral engagement, local investment and leadership will all enhance the resilience of conservation efforts in the face of future perturbations. Other actions aimed at enhancing resilience will require fundamental institutional change and extensive government and public engagement and support if they are to be realised.
5. The pandemic has highlighted the inherent vulnerabilities in the social and economic models upon which many conservation efforts are based. In so doing, it presents an opportunity to reconsider the status quo for conservation, and promotes behaviours and actions that are resilient to future perturbation.

A free Plain Language Summary can be found within the Supporting Information of this article.

## Abstract 2

A free Plain Language Summary can be found within the Supporting Information of this article.

**Postscript note**

It is with great sadness that we note the death of one of our co‐authors, Yunsiska Ermiasi, from COVID‐19 on 5 August 2021, 2 days before this paper was accepted for publication. Yunsiska was Deputy Director I, Operations, of Borneo Nature Foundation Indonesia, where she was responsible for establishing and overseeing numerous conservation projects, and was a true advocate for indigenous Dayak women in conservation. Aged only 40 and with so much still to give, her loss starkly illustrates the impacts of COVID‐19 in causing the premature death of conservationists and consequent psychological impacts of this on remaining colleagues.

## 1 INTRODUCTION

The COVID‐19 pandemic is restructuring our individual and collective behaviour on a global scale, including how we interact with each other, how and where we travel and how we work. While concerns about the societal impact of global pandemics have been repeatedly raised in the scientific and popular literature (e.g. Morens et al., 2010; Riva et al., 2014; Scanlon et al., 2007), many of us were left stunned by the rapidity and magnitude of societal change witnessed during 2020 and beyond. More than a year after the initial outbreak, uncertainties as to how COVID‐19 will continue to impact upon global society over the coming years and decades still remain (Walker et al., 2020). Amidst this uncertainty and fear, it is natural for conservation issues to drop off our collective radar. Indeed, in the early months of the COVID‐19 pandemic, a huge disparity in the media coverage of major conservation issues compared to the pandemic occurred (UNICEF, 2020). This is less the case today as science and the media increasingly highlight the perspectives that COVID‐19 sheds on conservation. These include the importance of healthy and intact ecosystems for reducing the risk of future pandemics (Carrington, 2020; Vidal, 2020), the significance of our interactions with the natural world for our health and well‐being (St‐Esprit McKivigan, 2020) and the potential for sectors and society to use our collective responses to the pandemic to rethink current unsustainable practices (Carpenter, 2020; De Bellaigue, 2020; Eisenstein, 2020).

If conservationists are to adequately respond to the social and ecological changes wrought by the COVID‐19 pandemic, they will need to rise to the funding, capacity and intensified environmental challenges the pandemic brings, as well as identify and act upon the opportunities it presents. In the medium to long term, conservation funding and government commitments are likely to be reduced by the societal restructuring and economic downturn that is underway (Corlett et al., 2020; Knight et al., 2020). In coming years and decades, any ongoing impacts of the COVID‐19 pandemic will be further compounded by the environmental, economic and societal changes that are predicted to occur as a result of global biodiversity loss and climate change (Kavousi et al., 2020). Compared to the pandemic, these changes may be observable at far slower rates, but will ultimately be more monumental and irreversible (IPBES, 2019; IPCC, 2018). Indeed, the emerging links between our changing climate, habitat degradation and fragmentation, biodiversity loss, increased human–wildlife interactions and incidences of novel zoonotic disease transmission (Carrington, 2020; Cheng et al., 2007; Grandcolas & Justine, 2020; Jones et al., 2008; Vidal, 2020) demonstrate that the need for effective conservation action is greater now than ever before.

The COVID‐19 pandemic presents fundamental challenges, as well as opportunities, for biodiversity conservation. The global reduction in human movement and economic activity resulted in a temporary lowering of carbon emissions, reductions in air, water, light and noise pollution and reduced wildlife disturbance as human populations retreated indoors (Kahn & Mehrotra, 2020; Schlichte, 2020). Yet, examples of new or enhanced pressures upon domestic animals and wildlife as a result of COVID‐related societal change are emerging, including increased hunting and harvesting pressure and the loss of food sources for wildlife (Ghosh & Aggarwal, 2020). The global reduction in mobility has had significant negative ramifications for the conservation sector, amidst the postponement or cancellation of research, monitoring and training/education programs and declines in travel and tourism revenue (Corlett et al., 2020; Evans et al., 2020; Harrison et al., 2020; Hockings et al., 2020). But the pandemic may also provide new opportunities for public engagement and research, with scientists calling for enhanced research efforts in the wake of the pandemic (Knight et al., 2020; Rutz et al., 2020).

In this paper, we draw upon examples from the peer‐reviewed literature to explore past societal, environmental and political perturbations and their outcomes for conservation. We detail the known and anticipated consequences of the current COVID‐19 pandemic for conservation, sourced from the emerging peer‐reviewed literature and six conservation case studies, which were drawn from our collective experiences as conservation scientists, practitioners and leaders from government and non‐governmental authorities, charities and research institutions. Using the findings from the literature review and these case studies, we aim to shed light upon: (a) the complex and often unanticipated implications of major perturbations, including COVID‐19, for biodiversity conservation; and (b) the potential opportunities presented by such perturbations for conservation monitoring, research and action.

## 2 METHODS

### 2.1 Identifying conservation outcomes of past perturbations and the COVID‐19 pandemic

Recognising the vast range of potential perturbations and related conservation outcomes, we chose to focus upon perturbations where major social, political, economic and/or ecological impacts would be quickly felt and readily ascribed to the perturbation in question. We thus excluded management decisions (e.g. whether to coppice a woodland, restore an ecosystem or to remove legal protections), and incremental changes such as gradual transitions from an industrial to service economy. Flooding and wildfire perturbations were excluded because, while several recent such events have been catastrophic for human and wildlife communities at the regional level, an initial scan of the literature highlighted the difficulty in disentangling frequent and less severe perturbations from more extreme events. We also excluded publications that measured biodiversity change along a gradient without explicitly linking this to conservation activities. We recognise our approach is not exhaustive, but believe it enables ready identification of a wide range of conservation impacts across different types of acute perturbation. Keywords incorporated the range of perturbations that were likely to have affected conservation sites around the world over the last century. Using the Web of Science Core Collection, the following search terms were entered on the 31 March 2021: TS = (biodiversity conservation AND (shock OR perturbation OR war OR volcan* OR eruption OR pandemic OR terroris* OR economic crash OR nuclear OR earthquake OR tsunami OR outbreak OR Ebola OR SARS OR MERS OR genocide OR Chernobyl OR Fukushima OR “Three Mile Island” OR Windscale OR swine flu OR “foot and mouth” OR zoonoses OR zoonotic OR “violent conflict” OR genocide OR Covid OR COVID‐19 OR coronavirus)).

From these results, all titles of English‐language publications published between 1900 and 2020 (*n* = 1,898) were read (see Nuñez & Amano, 2021 for limitations and biases of this approach). Abstracts were read in full when the title implied a focus upon a perturbation listed above, or biodiversity conservation activities. Full texts were downloaded and read (*n* = 168) in cases where the abstract described or hinted at observations relating to the outcomes of the perturbation on biodiversity conservation and conservation activities. Descriptive and quantitative information related to biodiversity conservation outcomes was then extracted from relevant publications, along with the type of perturbation and the continent where the research was primarily focused. We categorised reported biodiversity conservation outcomes into six categories: (a) wildlife, environment and ecology; (b) local income and livelihoods; (c) conservation activities, infrastructure and management; (d) conservation funding or income generation; (e) research foci; and (f) engagement and messaging.

### 2.2 Identifying conservation outcomes of COVID‐19 from case studies

Recognising that the COVID‐19 conservation research literature is only beginning to emerge, particularly in the context of conservation, we also assessed the known and anticipated outcomes of the COVID‐19 pandemic on conservation sites and species of conservation interest well known to members of our author team (i.e. at least one co‐author is based in the country or within the organisation and conducting conservation work). *Known outcomes* were categorised as those that had been observed by members of the author team, by a trusted or expert source or which had been extracted from written and verified documentation available from academic, government reports or grey literature (more detail on the individual case study methodologies in SOM 2). *Anticipated outcomes* were those that could not be independently verified or did not have observable impacts, but which were expected based upon local and author knowledge. Case studies aimed to complement and expand upon the literature review by highlighting specific and unpublished responses to the current pandemic. Case studies were selected based on co‐author expertise, with care taken to present a range of conservation settings, including terrestrial, coastal, public and private enterprises. We recognise our decision to utilise author expertise when choosing the case studies represent a specific bias in our methodology, hence our decision to use case studies and the emerging COVID‐19 literature to complement each other. Despite known biases, case studies describe the responses across a range of conservation settings, economies and cultures to the COVID‐19 pandemic. They aim to decipher the nuances and complexities of each case study, and highlight the known and foreseen short‐ and long‐term biodiversity conservation outcomes of the pandemic.

### 2.3 Identification of cross‐cutting issues and solutions for biodiversity conservation in the context of COVID‐19

From the wider COVID‐19 literature and the case studies, we extracted identified negative social, economic or ecological outcomes reported across multiple geographical and cultural contexts, and classed these as cross‐cutting conservation issues. Using the detailed case study examples, we identified specific impacts upon conservation emanating from these broader cross‐cutting issues, their likelihood of occurrence and anticipated duration. From the COVID‐19 literature and case studies, we then extracted suggested cross‐cutting solutions for biodiversity conservation in the face of ongoing or future perturbations, that is broad actions identified across multiple contexts as leading (or with the potential to lead) to improved conservation outcomes. We then extracted examples of how these solutions were, or could be, implemented or operationalised at the local level using our case studies.

## 3 RESULTS

Our search approach yielded a total of 111 papers that included descriptions of the biodiversity conservation outcomes of acute perturbations, including natural disasters such as tsunamis, earthquakes and volcanic eruptions (*n* = 12); warfare, violent conflict or civil strife (*n* = 35); technical perturbations such as large‐scale engineering projects or nuclear disasters (*n* = 6); sudden economic or political change (*n* = 8); and zoonotic disease outbreaks including COVID‐19 (*n* = 50). All continents except Antarctica came up in the literature review (see SOM 1 for the list of papers reviewed). The majority of these studies included observations on the direct impacts to wildlife, environments or ecological processes in the years to decades after a perturbation (*n* = 77), with studies also researching or discussing impacts upon local communities and livelihoods (*n* = 40), conservation actions including monitoring and infrastructure (*n* = 48), conservation funding and revenue (*n* = 16), suggested or observed changes to research foci (*n* = 18), and the impacts that engagement and messaging had upon conservation outcomes in the context of the perturbation (*n* = 22).

**FIGURE 1:**
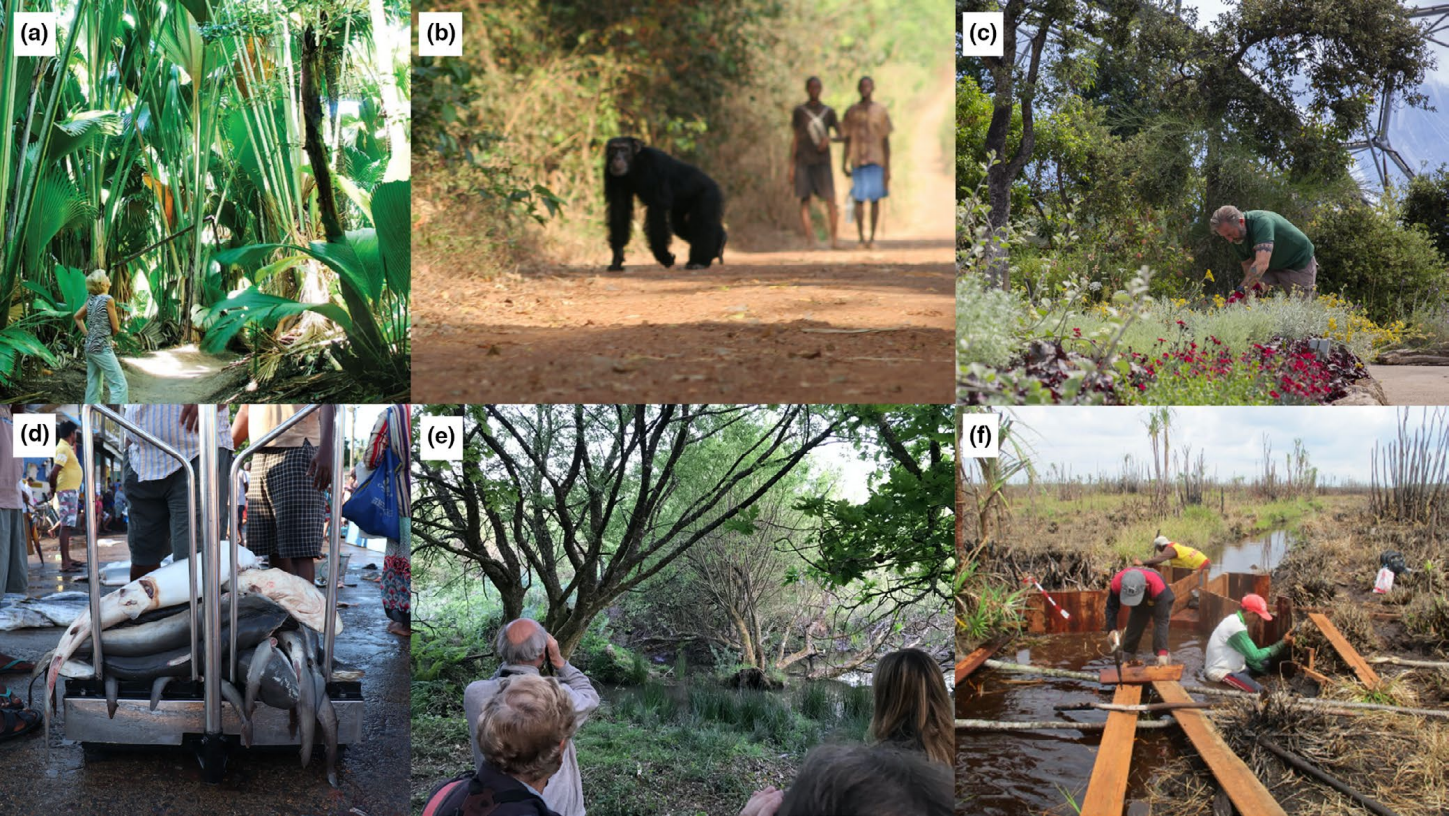
A conservation issue highlighted or driven by the COVID‐19 pandemic, by case study site. (a) In the Seychelles, international tourism temporarily ceased, meaning a complete loss of funds for many protected areas, including the UNESCO World Heritage site of the Vallée de Mai. (b) Chimpanzees and other wildlife may also be susceptible to the novel coronavirus, creating a possibility of inter‐species transmission especially in shared landscapes such as Cantanhez National Park, Guinea‐Bissau. (c) The Eden Project, UK, was closed to visitors for 75 days, with estimated revenue losses of up to 5 million GBP. (d) In Sri Lanka, sellers were quick to adapt to lockdown measures, but fishers, small‐scale traders and casual workers appear to have been the most impacted by the market changes resulting from the pandemic. (e) The Cornwall Wildlife Trust, UK, raises funds from community engagement events such as this public beaver walk, many of which were unable to occur in 2020. (f) In Indonesian Borneo, conservation actions such as habitat restoration (in this case, the damming of old illegal logging canals in the peat) have been able to largely continue because the work is managed and carried out by small local teams. Photograph credits: (a) Raymond Sahuquet, Seychelles Tourism Board, (b) Kimberley Hockings, (c) Eden Project Limited, (d) Claire Collins, (e) Cornwall Wildlife Trust and (f) Muhammad Idrus, Borneo Nature Foundation Indonesia. Where individuals are identifiable, consent has been gained for the use of their photograph for publication

Six contemporary case studies from geographically diverse conservation regions and sites provided detailed insights into the responses and conservation impacts related to the COVID‐19 pandemic. These included countries classed by the United Nations Human Development Index as having very high (the United Kingdom), high (the Seychelles, Sri Lanka and Indonesia) and low human development (Guinea‐Bissau; UNDP, 2019). The case studies spanned different geographical scales and governance and focused on COVID‐19 impacts on: biodiversity conservation at the country‐wide scale with a focus on the World Heritage sites (Seychelles case study, CS1); the running and governance of a national park (Guinea‐Bissau, CS2); the work and functioning of two charitable trusts (Eden Project, CS3, and Cornwall Wildlife Trust, CS5); the conservation outcomes for an industrial sector (Sri Lanka, CS4); and at a regional program level, with a focus upon the activities of a not‐for‐profit conservation organisation (Indonesian Borneo, CS6; Figure 1). Further details of each case study in the context of responding to the challenges of COVID‐19 for biodiversity conservation are provided in SOM 2.

### 3.1 The conservation outcomes of perturbations

#### 3.1.1 Wildlife, environment and ecology

The conservation outcomes of non‐COVID‐19 perturbations for wildlife, environment and ecologies are complex and vary greatly according to perturbation type, time since perturbation, species or habitat of interest, cumulative impacts of stressors and the social–cultural–political contexts involved.

##### Habitat degradation and fragmentation

Natural disasters, violent conflict and technological perturbations (e.g. building of energy infrastructure, nuclear accidents) commonly resulted in habitat degradation and fragmentation (Dudley et al., 2002; Hostert et al., 2011; Marske et al., 2007; Sayer et al., 2012). Natural disasters, in particular, caused immediate and extensive declines and alterations to habitats (Affan et al., 2019), in some instances paving the way for new habitats to form (Kurosawa, 2021). Habitat degradation was also commonly recorded as a direct result of war or violent conflict (Dudley et al., 2002; Nguyen, 2009), and occasionally from human responses to the threat of zoonotic disease (Olival et al., 2012). Some of the most extreme examples involved the wide‐scale destruction of highly diverse habitats via the drainage of wetlands (Olival et al., 2012; Richardson & Hussain, 2006), defoliation of forests (Nguyen, 2009) and the dumping of pollutants into aquatic environments (Lawrence et al., 2015). In such cases, habitats and their associated biodiversity take decades to recover, if they recover at all. Less extreme, but still significant, is the fragmentation and transformation of natural habitats into plantations or farmlands via the migration and settlement of populations displaced by conflict, or the incursion of roads and infrastructure by military or guerrilla groups (Butsic et al., 2015). Once conflict has subsided, the fragmentation of habitats and incursion of infrastructure often makes previously remote areas more accessible, opening up natural resource extraction opportunities and encouraging settlement of previously abandoned or remote areas (Dávalos, 2001; Clerici et al., 2020; Conteh et al., 2017; Enaruvbe et al., 2019; Grima & Singh, 2019).

While habitat fragmentation and loss are common outcomes of perturbation, those that result in the curtailment of human activity can create de facto wildlife refuges, at least during the course of the perturbation (Annecke & Masubelele, 2016; Calle‐Rendon et al., 2018; Coates, 2014; Constantinou et al., 2020; Conteh et al., 2017; Lawrence et al., 2015; Lindenmayer et al., 2016; Lindsell et al., 2011). Gains for conservation through de facto protection or land abandonment may, however, be negated by tactical habitat destruction or settlement of populations in highly diverse habitats elsewhere (Calle‐Rendon et al., 2018; Yong Sung et al., 2019). Long‐term refuges have also been created from changing political, economic and technical fortunes (Agyeman & Ogneva‐Himmelberger, 2009; Bragina et al., 2015; Hostert et al., 2011).

**COVID‐19:** The impacts of COVID‐19 restrictions on human movement and trade have had mixed impacts upon habitats to date, although quantifiable data are lacking. The decline in human movement has reduced travel to areas such as national parks, possibly reducing the pressure upon habitats that would normally sustain high volumes of traffic or human footfall (Miller‐Rushing et al., 2021). However, in locations where enforcement capacity was reduced or communities were driven to unsustainably harvest resources due to a loss of livelihood opportunities or declines in trade, habitat integrity was compromised (Lindsey et al., 2020, Schwartz et al., 2020; CS2, Table 1).

**TABLE 1:** Impacts related to biodiversity conservation, both observed and *anticipated* (in italics), across the six case studies as a result of the COVID‐19 pandemic

Impacts	Seychelles (CS1)	Cantanhez National Park (CS2)	Eden Project (CS3)	Sri Lanka (CS4)	Cornwall Wildlife Trust (CS5)	Central Kalimantan and BNF (CS6)
Wildlife, environment and ecology	◆ Pressure to import perishable goods by air resulted in several flights with new insect species in their holds, increasing biosecurity risks. ◆ Reduced disturbance led to less pollution and some rehabilitation of coastal vegetation. ◆ Increased illegal hunting incidents and pollution from illegal fires, with potential for escalation.	◆ *Possibility of inter‐species disease transmission including from humans to great apes directly or indirectly via transmission to other wildlife*. ◆ The economic downturn and 2020 cashew market collapse likely contributed to increased harvesting of forest resources for local consumption/sale.	◆ Increased reporting of wildlife in Cornwall and international wild sites, due to declines in disturbance.	◆ Loss of international trade in wildlife products of conservation concern has been severely disrupted. For example, shark fin sales have slowed amidst price drops of ~60%–70% and cessation of exports.	◆ Upon the initial reopening of nature reserves to the public in July 2020, CWT witnessed an upsurge in littering, non‐permitted activities and antisocial behaviour by a minority of visitors. ◆ During the initial 2020 lockdown period, CWT staff reported that seals were hauling out in locations that were normally too disturbed by human presence.	◆ *Possibility of inter‐species transmission including from humans to great apes directly or indirectly via other wildlife*. Safeguarding measures implemented and regularly reviewed. ◆ Impacts of COVID‐19 on local economic activities have led to some environmental improvements in the Sebangau watershed area, including reduced oil residues from boats and litter pollution in the river, *which may positively impact local river wildlife and fish populations, at least in the short term*.
Local income and livelihoods	◆ ~USD 3.8 m lost in cancelled visitor bookings (25 February 2020–23 March 2020) ◆ Decline in annual visitor arrivals of 70% between 2019 and 2020 (National Bureau of Statistics, Seychelles). ◆ Lost incomes for conservation and private organisations, for example lost revenue from Vallée de Mai UNESCO World Heritage site visits of ~USD 1.01 m. ◆ Drop in foreign exchange inflow of 62%, amounting to USD 221 m (Central Bank of Seychelles, Annual Report 2020). ◆ The UNESCO Heritage sites are run by the Seychelles Islands Foundation, loss of ~USD 1.13 m in 2020 compared to 2019.	◆ International collapse of cashew market in 2020 led to food insecurity: the loss of international markets led to many farmers selling their cashew harvest for 33%–75% lower price compared to previous years (UNDP, 2020). ◆ Increased food insecurity and reliance on natural resources including naturalised oil palm.	◆ Cessation of tourist income virtually overnight with estimated revenue losses of GBP 4.5–5 m. ◆ >350 staff members placed on the UK government furlough scheme.	◆ Fishers, small‐scale traders and casual workers primarily affected. Initial price reductions of ~50%–60% for fishers at landing sites and cessation of casual, market‐based work. ◆ The price of fish at landing sites declined dramatically for high‐value fish (e.g. tuna) sold fresh domestically or exported, but not for shark (dried for domestic market). ◆ Rise in consumer prices (~50%–60%) likely due to demand, loss of traditional sales methods, potentially leading to food insecurity.	◆ A third of staff were placed on administrative leave and volunteers stood down from March 2020. ◆ Compared to the same period in 2019, the income from new memberships between March and May 2020 declined by 57%, with government support not sufficient to make up for overall income reduction. These factors have reduced the CWT’s ability to carry out key activities and commitments outlined in their business plan.	◆ Negative impacts on the economy of local community members in Sebangau for whom tourism is important, including for those working as tour guides, tour operators, and in transportation and consumption services. ◆ Local communities experience wider socio‐economic disruptions, including school closures and limited work opportunities, *likely impacting women and informal workers the most*. ◆ To overcome *potential consequent negative impacts on forest areas*, BNF has increased activities with partners to involve more community groups in the local patrol activities in the part of Sebangau where it focuses its research, and to support community groups in seedling nursery development, planting and permaculture training.
Conservation activities, infrastructure and management	◆ Discontinuation of key biodiversity monitoring at sites across the country. Some remote islands, for example Aldabra, were sufficiently isolated to continue routine monitoring. ◆ Delays in or reduced levels of invasive alien species control. ◆ Delays to essential repairs and maintenance to support conservation, compromising safety of staff working in remote locations.	◆ Continued monitoring work will enable identification of possible COVID‐19 impacts, such as increased reliance on forest resources, including bushmeat and timber. ◆ *Presence of park guards may discourage illegal activities in protected zones*. ◆ Guards and researchers trained in use of protective equipment to reduce human‐to‐wildlife COVID‐19 disease transmission.	◆ Skeleton staffing of four horticulturalists for the Eden estate, with overgrowth and damage to rare plant collections observed. ◆ Reduced monitoring and surveying efforts as science team was locked down. ◆ Demonstration of Eden's core themes including the importance of wild spaces for health.	◆ *Diversion of efforts from management and regulatory enforcement to supporting distribution of fish may have facilitated illegal activities. Fishers reported landing of prohibited species, while multi‐day vessels have been arrested during the pandemic for fishing illegally in other countries waters. However, more research is needed to ascertain to what extent these actions were influenced by changes in monitoring and enforcement*.	◆ *Long‐lasting implications on how staff work and travel to conservation sites, and ongoing requirements to balance caring responsibilities, social distancing and shielding of vulnerable people, are expected. This is particularly pertinent to the CWT as a significant proportion of their volunteers, trustees and members are >70 years of age or classed as vulnerable*.	◆ Habitat conservation and restoration efforts have continued with modifications as they involve small numbers of people working in areas away from human populations. ◆ *Firefighting may become more difficult during the pandemic, as frequently involves large teams*. ◆ Activities that can be conducted by individuals and small (socially distance‐able) teams have been impacted relatively little, but travel and goal/target setting (particularly for non‐forest‐based work) has been complicated. The pandemic has impacted community, government and other third party in‐person meetings and liaison, particularly those involving large groups of people. ◆ Difficulties in M&E implementation for social research/projects (education, community development), including through online requests.
Conservation funding and income generation	◆ Loss of international research expertise with many research trips cancelled. ◆ Cancellation of funded conservation projects—unknown if funds for existing projects will remain available after the COVID‐19 pandemic. ◆ Uncertainty over future conservation priorities and funding. ◆ Opportunities for small‐scale international funding as rapid response to COVID‐19 situation.	◆ Cessation of international research travel resulting in delays to biological and social data collection, including conservation‐related activities, with some follow‐on impact to livelihood opportunities. ◆ Reduced capacity to plan and apply for funding for future research. ◆ Uncertainty over future conservation funding.	◆ New projects—the establishment of new Eden Projects in the UK and internationally were impacted as a result of the team being unable to travel. Any time lost through the pandemic is expected to be regained in 2021. The majority of the projects have remained on‐track. The biggest impact was the delay to the opening of the Dubai Expo 2020 by a year to 2021	◆ Collection of landings data of small‐scale fleets has continued; however, planned improvements for multi‐day vessel landings, including physical verification at landing sites, have temporarily ceased. Data collection relies on accuracy and honesty within log‐booking reporting, which is sometimes compromised.	◆ Cancellation of community and fundraising events has impacted engagement and membership recruitment. Previously, membership and fundraising were achieved through CWT attendance at events and festivals, as well as door to door, but most 2020 events have been cancelled, shifted online or are deferred.	◆ Most of BNF’s forest‐based research involves small teams and has continued with modification. Temporary suspension of primate behavioural ecology field research to reduce the chances of inter‐species COVID‐19 transmission (under review at time of writing). ◆ Community‐based research plans re‐assessed and in some cases delayed, international research visits suspended (ongoing at time of writing) and some field projects cancelled. ◆ Renewed prioritisation of local partnerships and strengthening of local field research teams. ◆ Some negative impacts on BNF’s income generation have occurred and are likely to continue in the short to medium term. ◆ Opportunities to deliver online courses, environmental education sessions and conservation webinars.
Research foci	◆ *Potential for redirection of conservation funds towards COVID‐19‐related issues at the cost of conservation projects*.	◆ *Potential for increased focus upon inter‐species disease transmission and risk, including building on existing research to include COVID‐19 in project objectives*		◆ *Potential for increased focus upon dynamics of social–ecological systems, responses to shocks and fisheries management under uncertainty*.		◆ *Potential for increased focus on existing datasets, given increased difficulty in initiating new research projects*. ◆ *Opportunities for new research projects and funding on mitigating COVID‐19 impacts*. ◆ *Opportunities for strengthening roles of local researchers within international collaborations, and empowering local research teams*.
Engagement and messaging	◆ *Compromised partnerships leading to potential erosion of trust and exchange between local and international partners, resulting in a loss of connectivity and knowledge‐sharing*. ◆ Increased online opportunities to connect for workshops and general knowledge exchange, for example Marine World Heritage Managers' forum.	◆ Renewed emphasis on the importance of in‐country collaboration across government, conservation organisations and research institutes to continue conservation and research activities. ◆ Engagement with local communities prioritised COVID‐19 health messaging with conservation participatory activities reduced ◆ Development of education materials for tourists and guides to prevent disease transmission to great apes and local communities. ◆ Renewed prioritisation of local partnerships and strengthening of local field research teams.	◆ The lockdown has led to a greater level of innovation and trialling of new digital content. This includes the development of the Eden Universe scheme and the installation of a 5G network on the Eden site and 360 degree cameras of develop a series of AR and VR activities to help drive new audiences on site. ◆ Enhanced attention on charitable mission and messaging during lockdown. For example, efforts to reduce loneliness, restoration of Cornish black bee populations.	◆ Importance of advancements in monitoring and data collection emphasised. Facilitated by continued emphasis on investing in expertise and skills of local scientific talent and infrastructure.	◆ Refocusing of priorities and development of a recovery plan. In the short term, this includes the focusing of public‐facing communications on the health and well‐being effects of nature. *Longer term this will include a review of strategic priorities via a recovery plan*.	◆ Temporary suspension of in‐person group activities (e.g. children's education, village training and Community Development events). Some of these have been possible to partially mitigate through alternative (virtual) approaches or to resume recently with smaller groups/mitigation measures, often resulting in reduced effectiveness. Forest‐based school visits and university field courses postponed or cancelled. ◆ Opportunity to highlight the importance of environmental conservation for public health, especially regarding reducing wildfire incidence. *Increased opportunities and support from public health agencies*. ◆ *Could increase support for programs with a positive impact on local communities, including alternative livelihood development*.

##### Changes to community assemblages and ecological processes

Assemblage and ecological effects from perturbation are difficult to demonstrate due to the lack of long‐term monitoring studies and the time it takes for changes to become apparent (Irving et al., 2018; Nguyen, 2009; Richardson & Hussain, 2006). A persistent decline in wildlife abundance and species richness was observed in the eastern Kratie province of Cambodia over a 50‐year period of extended conflict, which was consistent with the proliferation of arms, the emergence of an external wildlife trade and Khmer Rouge‐era government policies that mandated hunting (Loucks et al., 2009). Wildlife behaviour may also be altered by attempts to control disease transmission between wild and domestic animal or human populations (e.g. erection of fencing to halt the natural migration of large ungulates, De Vos et al., 2016), with corresponding impacts upon ecological processes. In the case of pollutants spread by aquatic and airborne pathways, ecological processes may be impacted across far larger scales than the initial perturbation (Mehli et al., 2000). For example, the 2011 Japanese Fukushima Daiichi nuclear accident resulted in the leakage of radionuclides directly into the ocean and adjacent land (Hiyama et al., 2012; Murase et al., 2015), while migrating bluefin tuna were shown to carry high levels of radionuclides from Japanese waters across the entire Pacific Ocean (Madigan et al., 2012).

**COVID‐19:** While the effects of the COVID‐19 lockdowns upon biodiversity are still emerging, anecdotal evidence has demonstrated both positive and negative outcomes upon ecological processes (Cheval et al., 2020). Reduced economic activity led to (unquantified) improvements in the Sebangau watershed in Central Kalimantan, including reduced pollution (CS6, Table 1). Reductions in human activity allowed wildlife to exploit new habitats or move back into areas that were previously abandoned (Waithaka et al., 2021, CS1, CS3, Table 1), leading to increased species richness in some areas, at least temporarily (Manenti et al., 2020). Some protected areas also reported fewer disturbances of animals due to lower visitation rates, and fewer incidences of road kill (Smith et al., 2021). However, enhanced illegal trafficking of wildlife was reported in some regions as livelihoods collapsed or enforcement efforts were reduced (Cherkaoui et al., 2020; CS1, Table 1).

##### Species persistence

Perturbations impact species persistence via the degradation of habitat, direct exploitation or persecution, or changes to ecological processes (Bragina et al., 2015; Hilton et al., 2003, Hirayama et al., 2020; Reynolds et al., 2017; Steutermann Rogers, 2018; Zhang et al., 2009). In areas of conflict, the introduction of military infrastructure, defoliation, the exploitation and trade of wildlife by displaced people, or military or insurgent armies for food or to finance war efforts can have devastating effects upon species abundance (Brito et al., 2018; Butsic et al., 2015; Draulans & Van Krunkelsven, 2002; Dutta, 2020). The persistence of pollutants, residues and abandoned munitions from conflict and technological perturbations, while potentially reducing the rate of encroachment by human populations, can cause considerable mortality to wildlife (Dávalos, 2001). Wildlife populations can also be put at risk from the transmission, or perceived risk of transmission, of zoonotic diseases to domestic animals or human communities, while transmission‐induced directed culling of wildlife may lead to local extirpation (Bicca‐Marques & Santos de Freitas, 2010; Donnelly et al., 2003; Walker & Nadin, 2011). Significantly, impacts may be felt for decades after the perturbation, impacting the future conservation potential of sites (Dudley et al., 2002; Møller & Mousseau, 2007a, 2007b; Richardson & Hussain, 2006).

**COVID‐19:** The loss of livelihoods and food insecurity has resulted in some communities increasing their exploitation of wildlife and habitats, including the illegal exploitation of species within protected areas (Phua et al., 2021, CS1), and species at high risk of extinction (e.g. Pinder et al., 2020; Yang et al., 2020). The persecution (illegal or otherwise) of wildlife perceived as pests, including endangered species such as wolves and raptors, has also reportedly increased, although quantifiable data are lacking (Cherkaoui et al., 2020). Species susceptible to COVID‐19 transmission, such as great apes, are at greater risk of contracting the disease from visitors unless strict safeguarding measures are implemented, with unknown but possibly severe consequences for population persistence (CS2, CS6, Table 1). Scientists have called for the strengthening of wildlife trade regulations to close loopholes in current governance to reduce the risk of zoonosis emergence, and the need to balance biodiversity conservation with the protection of food security and livelihoods of communities dependent on this trade (e.g. Booth et al., 2021; Borzée et al., 2020; Roe et al., 2020).

#### 3.1.2 Incomes and livelihoods

Changes to local incomes and livelihoods as a result of perturbation have major implications for biodiversity conservation. When poverty, unemployment and social instability suddenly escalate, local and global demand for natural resource consumption can alter significantly (Grima & Singh, 2019; Sayer et al., 2012). Natural disasters, war and civil strife, for example, can accelerate fragmentation of habitats or declines in species abundance as displaced people hunt, convert or harvest resources in what were previously remote or unexploited areas (Butsic et al., 2015; Conteh et al., 2017; Dudley et al., 2002). Increased hunting pressure and shifts in trading patterns can result in enhanced trading of endangered species (Loucks et al., 2009; Zahler et al., 2004). Conversely, human movement or decreased accessibility due to, for example, violent conflict may reduce the rates of land conversion or extractive industries in previously worked areas, enabling recovery of habitats (Gaynor et al., 2016).

Efforts to reduce disease transmission between wildlife and domestic animals can result in measures that directly and indirectly impact local incomes and livelihoods by adding administrative hurdles and additional costs to businesses and individuals (De Vos et al., 2016). In areas where protected areas occur adjacent to domestic farms, disease transmission can negatively impact the economic earnings of local communities when livestock have to be slaughtered or are unable to be marketed due to disease (De Vos et al., 2016; Wu & Perrings, 2017). Perturbations can also lead to a sudden and sustained decline in tourist numbers, impacting local revenue and livelihoods (De Vos et al., 2016; Gardner et al., 2016; Santos, 2020). The loss of income from tourism can impact biodiversity by increasing peoples’ reliance upon natural resources as incomes suffer, and by reducing incentives to conserve wildlife and habitats central to tourism‐based industries (Gaynor et al., 2016).

**COVID‐19:** In Guinea‐Bissau, the COVID‐related collapse of the international cashew market in 2020 led to food insecurity for farmers working in the Cantanhez National Park (CS2, Table 1). Similarly, a loss of export trade in Sri Lanka meant price reductions of landed fish, particularly in high‐value species, as well as the cessation of casual, market‐based work (CS4, Table 1). A drop in tourism visitation also has implications for local livelihoods, including paying the wages of conservation and protected area personnel such as guards and tour guides, and those employed by the wider tourism industry (Smith et al., 2021; CS1, CS3, CS5, CS6, Table 1). A decline in tourist arrivals into the Seychelles of 70% was seen in 2020, with the loss of revenue for tourism and protected area sites in the order of millions of USD (CS1, Table 1).

#### 3.1.3 Conservation activities, infrastructure and management

The capacity of conservation organisations to continue engagement activities, research, monitoring and maintain networks and infrastructure is often curtailed in the face of acute perturbations. In the cases of war or strife, the deterioration of stability may lead to an increase in violence, illegal hunting and illicit trade, placing additional pressure upon conservation organisations and putting workers’ lives at risk (De Merode et al., 2007; Draulans & Van Krunkelsven, 2002; Gaynor et al., 2016). In such cases, conservation organisations may withdraw funding and workers from a region, disrupting conservation progress and the relationships built over the years with local communities (Hart et al., 1997). In warfare and disaster zones, areas may be dangerous and difficult to access for months or years after the event, with conservation infrastructure and networks damaged or lost (Zhang et al., 2014). Such capacity can take a long time (years or decades) to rebuild (Conteh et al., 2017), while the cessation of monitoring activities reduces the evidence base of resulting biodiversity change, with corresponding knock‐on effects for decision‐making (Hilton et al., 2003).

Post‐disaster reconstruction activities can be directed to benefit biodiversity, particularly when the restoration of highly diverse habitats helps reduce the impact of perturbations, or when ‘grey’ infrastructure is replaced with ‘greener’ infrastructure (Kurosawa, 2021). However, communities and governments in crisis mode are unlikely to prioritise environmental protection unless environmental governance is particularly resilient or conservation organisations work in concert with existing social and cultural institutions and development/reconstruction goals (De Merode et al., 2007; Hart et al., 1997; Kurosawa, 2021; Main & Dearden, 2007). The use of military personnel, infrastructure and partnerships to conduct conservation activities has also been employed to achieve conservation outcomes post‐perturbation. However, ‘militarised conservation’ can have severe societal and psychological repercussions, particularly for communities previously traumatised by war (Dutta, 2020).

**COVID‐19:** The COVID‐19 pandemic disrupted or entirely halted the activities of conservation programs throughout 2020 and 2021, including interrupting routine management and monitoring activities (Cheval et al., 2020; Manenti et al., 2020; Sugai, 2020), although some activities were permitted to continue in areas away from human populations (CS1, CS6, Table 1). In the Seychelles, lockdown hampered conservation activities aimed at controlling invasive alien species, with potential negative impacts upon native wildlife (CS1). Parks and conservation organisations that stayed open had to deal with additional complications of reduced capacity and/or increased costs of implementing additional safety procedures, including staff absence and the need for social distancing measures when housing workers and conducting routine conservation tasks (Miller‐Rushing et al., 2021; CS1‐CS6, Table 1). These difficulties meant that less urgent tasks—including research projects and long‐term monitoring—were not prioritised (Miller‐Rushing et al., 2021; CS1‐CS5, Table 1).

Despite the many negative effects of COVID‐19, the pandemic also provided an opportunity for organisations to consider paradigm shifts towards more interdisciplinary, inclusive and equitable conservation (Roe et al., 2020). The lockdown provided an opportunity for the Eden Project, in Cornwall, to demonstrate its core themes, including the importance of wild spaces for physical and mental health (CS3). Researchers have also called for greater interconnectedness between economic and ecological restoration policies as a way to reverse biodiversity loss while providing livelihood opportunities for millions of people (Singh et al., 2020).

#### 3.1.4 Conservation funding and income generation

The type and quantity of funding available for conservation is impacted by perturbation events in multiple ways. The outbreak of conflict, or major social–political shifts can result in the withdrawal of conservation funding (Hart et al., 1997). The severing of international development aid has been shown to negatively impact conservation activities in lower‐income countries, particularly if the in‐country regime holds different priorities to its former donors, or if corruption is rife (Hart et al., 1997). Withdrawal of funding can result in a total loss of conservation capacity, unless partnerships between conservation organisations, environmental institutions and local communities are particularly resilient (Hart et al., 1997; Newton, 2011). Conversely, natural disasters may result in increased funding for conservation‐related activities where reconstruction efforts focus upon restoring natural habitats as buffer zones (Strusińska‐Correia, 2017). The declarations of disease presence in a region, such as malaria, may have significant implications for tourism numbers and revenue (De Vos et al., 2016). Conversely, prevalence or introduction of diseases in local wildlife, or threat or transmission to domestic animals or human communities can also stimulate funding for research into reservoir species or transmission pathways (De Vos et al., 2016).

**COVID‐19:** The impacts of COVID‐19 were immediately felt by tourist destinations and conservation programs, including World Heritage Sites and protected areas, that depended upon tourism and visitor revenue to finance conservation infrastructure, monitoring, research, engagement activities and personnel (Bhammar et al., 2021; Lindsey et al., 2020; Miller‐Rushing et al., 2021; Waithaka et al., 2021, CS1, CS3, CS5). While future revenue may be recouped by return to normal levels of travel and activity post‐pandemic, further disruptions, or future recession and austerity measures are likely to magnify the impact on conservation programs if tourism declines, or funding is reduced or redirected towards humanitarian aid or economic relief (Cumming et al., 2021; Evans et al., 2020; Kavousi et al., 2020; Lindsey et al., 2020). Many conservation programs and protected areas around the world are already chronically underfunded (Phua et al., 2021), and further shortfalls from the loss of traditional revenue streams may negatively impact the delivery of conservation outcomes (Bhammar et al., 2021; CS1‐CS3, CS5, CS6, Table 1). These impacts will be amplified if decision‐makers and funders choose to prioritise ‘business as usual’ above longer term biodiversity goals as the world emerges from pandemic restrictions (Cheval et al., 2020; Corlett et al., 2020). The impact of the pandemic will also spill over into future project planning, as proposed projects are likely to be scaled back or delayed while funds are tight. For example, the establishment of new Eden Projects in the United Kingdom and internationally were impacted as a result of the project team not being able to travel, although the time lost during the pandemic is expected to regained during 2021 (CS3, Table 2).

**TABLE 2:** Cross‐cutting issues and specific impacts of COVID‐19 on conservation, their likelihood and duration, across the six case studies. YES/Green colour = Impact is known to be occurring. POSSIBLE/Orange colour = Impact could potentially occur but has not yet been observed by the authors. UNLIKELY/Red colour = Impact is considered unlikely to occur in the short or long term. Colour gradation represents the duration of the impact: the darker the colour, the longer the impact is likely to continue

Cross‐cutting issues	Specific impacts on conservation	Seychelles (CS1)	Cantanhez National Park (CS2)	Eden Project (CS3)	Sri Lanka (CS4)	Cornwall Wildlife Trust (CS5)	BNF, Borneo (CS6)
Reduced funding or income	Reduction in tourism income leads to loss of funds for conservation activities and local communities	YES	YES	YES	NA	YES	YES
	Increased reliance on local resources due to loss of livelihoods	POSSIBLE	YES	NA	POSSIBLE	UNLIKELY	POSSIBLE
	Increased illegal hunting and other illegal activities	YES	POSSIBLE	NA	POSSIBLE	UNLIKELY	POSSIBLE
Lack of data	Discontinuation or suspension of key monitoring	YES	YES	YES	YES	POSSIBLE	YES
Loss of partnerships	Erosion of local partnerships and/or trust between researchers and local communities	POSSIBLE	POSSIBLE	UNLIKELY	UNLIKELY	POSSIBLE	POSSIBLE
Reduced local capacity/fewer opportunities for enhancing capacity	Reduction in local conservation capacity for monitoring and research	YES	POSSIBLE	UNLIKELY	UNLIKELY	UNLIKELY	POSSIBLE
International withdrawal	Cancellation of research and conservation projects	YES	YES	POSSIBLE	YES	POSSIBLE	YES
	Redirection of funds away from conservation activities	POSSIBLE	YES	POSSIBLE	POSSIBLE	POSSIBLE	POSSIBLE

#### 3.1.5 Research foci

Priorities for future research post‐perturbation varied according to the perturbation and disciplinary focus of the researchers. Across perturbation types, calls were made for more interdisciplinary research and enhanced partnerships with local institutions, development agencies and regional/national government, with the aim of finding innovative ways of dealing with complex conservation challenges resulting from the perturbation (Butsic et al., 2015; Brito et al., 2018; Calle‐Rendon et al., 2018; Conteh et al., 2017; Dudley et al., 2002). Research into zoonotic disease called for holistic approaches to understand the links between zoonotic transmission, public health and biodiversity loss (Cunningham et al., 2017; De Vos et al., 2016; Wood et al., 2017), while studies of conflict‐hit regions highlighted the need to understand not only the opportunities but also the challenges that peace and rebuilding bring for conservation and natural resource management (Grima & Singh, 2019; Zúñiga‐Upegui et al., 2019). Greater understanding as to which factors contribute to the resilience of conservation management, infrastructure and community networks in the face of perturbation was also called for (De Merode et al., 2007; De Vos et al., 2016).

**COVID‐19:** Researchers expressed concern that biodiversity conservation will not be seen as a funding priority post‐pandemic (Corlett et al., 2020; Ramvilas et al., 2021, CS1). Yet, the pandemic has also brought the need for particular research topics into focus. The importance of understanding the global wildlife trade and its links to zoonotic transmission and biodiversity loss has been highlighted since the emergence of the COVID‐19 pandemic (Booth et al., 2021; Borzée et al., 2020; D'Cruze et al., 2020). Calls for holistic approaches such as the One Health approach, that recognise the complex interactions between human and wildlife, how such interactions drive disease transmission and biodiversity loss, and better understanding of the public health consequences of biodiversity loss have been made (Calistri et al., 2021; Campos & Lourenço‐de‐Moraes, 2020; Harrison et al., 2020; Ramvilas et al., 2021; Terraube & Fernández‐Llamazares, 2021, CS2). Calls have been made to refocus conservation research agendas and recognise inevitable trade‐offs in the light of reduced future spending and accelerating climate and biodiversity crises (Kavousi et al., 2020). A better understanding of human–nature interactions, including inter‐species disease transmission pathways, the opportunities and motivations behind people seeking (or not seeking) interactions with nature and how motivations have been altered by the pandemic has also been called for (Soga et al., 2021, CS2).

#### 3.1.6 Engagement and messaging

The devastation caused by natural disasters has prompted communities and national governments to initiate restoration of habitats that form natural barriers against storm and surge damage (Affan et al., 2019; Barbier, 2006). Species exploitation may decline if the possibility of zoonotic disease infections is perceived or known by the community (Gbogbo & Kyei, 2017), or stricter enforcement measures, transport and trading bans are implemented (as in the case of SARS‐CoV, Bell, 2004). Awareness campaigns can also play a critical role in protecting biodiverse habitats and wildlife (Montana & Mlambo, 2019). Conversely, fear of disease transmission can lead communities to exterminate local populations of species associated with the disease (Bicca‐Marques & Santos de Freitas, 2010), or come to resent management measures (such as protected areas or game reserves) that support or protect species associated with disease transmission (De Vos et al., 2016).

**COVID‐19:** Publications highlighted the importance of generating messaging that effectively demonstrates the linkages between conservation, intact and healthy habitats; human well‐being; pandemic risk and inter‐species transmission; and the climate and biodiversity crises (Corlett et al., 2020; Evans et al., 2020; Harrison et al., 2020; Laffoley et al., 2020; Lu et al., 2021; MacFarlane & Rocha, 2020). Rangers working within the Cantanhez National Park, Guinea‐Bissau, were trained in how to reduce inter‐species disease transmission and risk (CS2). Both the Eden Project and Borneo Nature Foundation saw the COVID‐19 pandemic as an opportunity to increase attention to messaging about the importance of species/habitat restoration and alternative livelihood development (CS3, CS6, Table 1).

The cancellation of in‐person meetings and conferences across industry, government and academia is likely to lead to key developments and decisions being postponed (Corlett et al., 2020). Concerns have been raised that the impact of cancelled field trips, meetings and other networking or skill‐based opportunities may further entrench existing biases within conservation (Harrison et al., 2020). On the other hand, researchers and conservation practitioners have argued that online meetings are potentially more equitable and inclusive as researchers and conservation staff have greater opportunity to attend (Miller‐Rushing et al., 2021, CS1), and that online platforms provide opportunities to expand public engagement through, for example, citizen science programs (Kishimoto and Kobori, 2021; CS3).

The engagement of local communities in conservation may also suffer as field and education activities and collaborations are halted or restricted (Lindsey et al., 2020, CS1, CS6), although technology has played a role in maintaining engagement in some communities as well as providing opportunities to test new approaches to engagement (Harrison et al., 2020; Miller‐Rushing et al., 2021, CS6). Loss or reduction of in‐person engagement mechanisms may have longer term effects if remote communities become increasingly wary of visitors bringing disease, while trust may decline if long‐term relationships are put on pause (Harrison et al., 2020). In some field sites, however, the pandemic provided an opportunity to renew the emphasis on in‐country partnerships and collaboration (CS2, CS6), and to enhance public engagement related to local wildlife and biodiversity issues (CS3, CS5).

### 3.2 Cross‐cutting issues for biodiversity conservation as identified from the COVID‐19 pandemic

**FIGURE 2:**
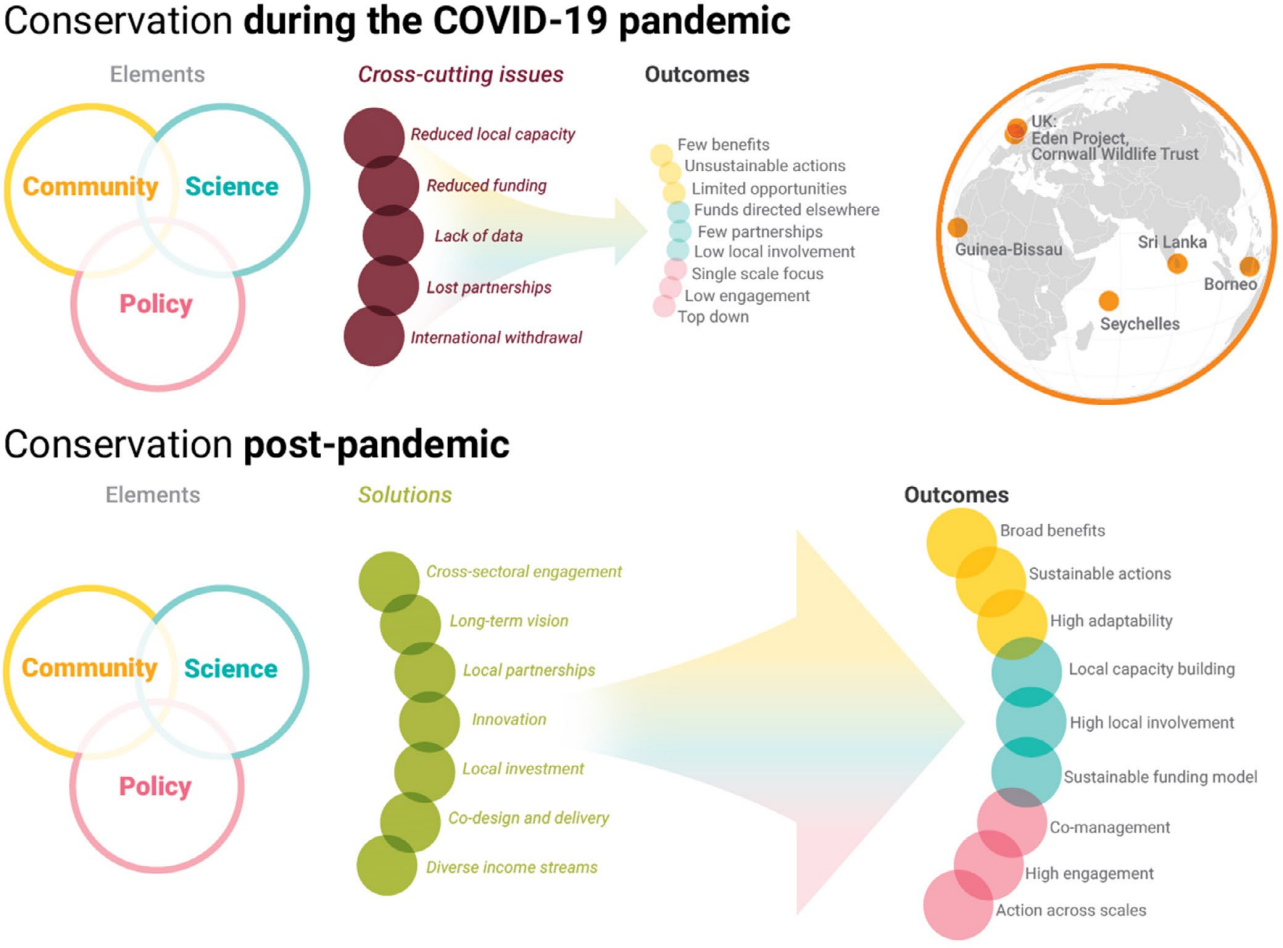
Delivering biodiversity conservation post‐pandemic. Conservation comprises multiple overlapping *elements*, which can be broadly and loosely defined by ‘communities’ (human: local, national and international; and ecological), ‘science’ and monitoring, and ‘policy’ or management. Biodiversity conservation is currently impeded by a number of *cross‐cutting issues* caused or exacerbated by the COVID‐19 pandemic, that negatively impact the delivery of conservation goals leading to suboptimal *outcomes*. If we are to promote positive outcomes for conservation in a future where the rate and scale of perturbations is likely to increase, we must chart a course for change. A set of *solutions*, synthesised from our case study experience (see map inset) and already adopted in some areas, could achieve positive *outcomes* for communities, science and policy, and achievement of conservation goals. Illustration by Nigel Hawtin

We identified five cross‐cutting conservation issues that were being caused, or were likely to be exacerbated by the COVID‐19 pandemic (Table 2; Figure 2). These were: (a) *reduced funding and/or income* resulting from lowered visitation/memberships that directly contributed towards conservation activities (Lindsey et al., 2020; Smith et al., 2021; CS1, CS2, CS3, CS5 and CS6) or which provided income and livelihoods to local communities (CS2 and CS6), and concerns that international funds would be reduced or redirected elsewhere (Cheval et al., 2020; Corlett et al., 2020; CS1–6); (b) a reduction in or *lack of monitoring data* caused, or exacerbated, by closures and social distancing restrictions (Cheval et al., 2020; Sugai, 2020; CS1, CS3, CS4 and CS6); (c) *loss of partnerships* had not yet been observed in our case study sites, but were of concern to local experts (CS1, CS2, CS5 and CS6); (d) *reduced local capacity* due to travel and work restrictions and lost training opportunities (Miller‐Rushing et al., 2021; Phua et al., 2021; CS1, CS2, CS4 and CS6); and (e) *withdrawal of researchers and practitioners* from conservation sites due to travel restrictions, which led to the cancellation or postponement of research (CS1–4 and CS6; Table 2).

### 3.3 Solutions and opportunities for improving biodiversity conservation as identified from the COVID‐19 pandemic

We identified seven solutions that—if implemented—would likely result in positive outcomes and promote opportunities for biodiversity conservation, in turn minimising the impact of future perturbations on conservation activities (Figure 2). Examples from our case studies highlighted how such solutions could be (or were already being) implemented (Table 3). (a) Having a clear *long‐term vision* was considered key to anticipating the ways in which conservation action and messaging could be continued and amplified post‐pandemic (e.g. identifying and implementing likely long‐term social and economic changes to implement sustainable work and fundraising models and conservation‐facing activities, CS1, CS3, CS5 and CS6, and promoting the need for systemic change in the ways we interact with nature, CS3). The need for clear priorities to make trade‐off decisions easier when conservation capacity is suddenly limited was also mentioned (Miller‐Rushing et al., 2021). (b) *Cross‐sectoral engagement* was regarded as necessary to identify and respond to ongoing and newly identified conservation needs (e.g. working across supply chains to understand the direct and indirect effects of lockdown and the potential consequences for conservation, CS4, and recognising and embracing the need for new training and knowledge generation, CS2 and CS6). (c) The creation and strengthening of *local partnerships*, (d) *co‐design and delivery* of conservation; and the need for (e) *local investment* and *leadership* were highlighted as important to ensure that effective conservation continues when capacity declines (CS1, CS2, CS5 and CS6). This includes efforts to enhance messaging about the significance of biodiversity conservation for human well‐being and livelihoods (Laffoley et al., 2020), finding ways to incentivise greater community participation across conservation programs and research (Cherkaoui et al., 2020; Ramvilas et al., 2021) and promote sustainable tourism and funding models that do not rely on large visitor numbers and/or which maximise local benefits (McGinlay et al., 2020). (f) The importance of *diverse income streams* to protect conservation funding and local livelihoods was highlighted across case studies (CS1, CS2, CS3, CS5 and CS6; Table 3). The literature also highlighted routes to the diversification of income streams via the development of virtual tourism opportunities, the creation of sustainable finance models such as selling of carbon offset credits, crowdsourcing, attraction of private or philanthropic capital and microfinancing initiatives (Cherkaoui et al., 2020; Cumming et al., 2021; Lindsey et al., 2020; Phua et al., 2021; Smith et al., 2021). (g) Finally, *innovation,* in terms of adopting new technologies and techniques to engage the public, maintain tourism (income), deliver conservation messaging and ensure the continuation of research and monitoring—as well as identifying new avenues of research—was seen as key to reducing the impacts of similar future perturbations on conservation activities (CS1–CS6; Figure 2). This also included suggestions to adopt innovative technologies such as artificial intelligence and machine learning to enhance data collection and processing abilities (Ramvilas et al., 2021).

**TABLE 3:** Implementation of solutions for biodiversity conservation in the wake of the COVID‐19 pandemic from case studies (numbered)

Cross‐cutting solution	Example	Explanation	Implementation
Long‐term vision	Opportunities for systemic change	Measures and ideas for fundamental system change existed pre‐pandemic, but have not yet been applied at the breadth and scale needed	The pandemic has cogently demonstrated how quickly large‐scale regulatory and behavioural changes can occur, and the interconnectedness of our actions and outcomes for public health. These provide opportunities to build support for systemic change at the grassroots level (CS3, CS6)
	Elevating the importance of biodiversity conservation in political agendas	The renewed interest in conservation, regeneration, agronomy, food security and the green economy may force these issues to rise up political agendas. If so, conservation organisations are in a strong position to offer education, consultancy and advice, with increased research and collaboration opportunities	National Parks and businesses such as the Eden Project (CS3) could form a natural testbed for understanding human‐natural capital, the health and well‐being effects of the natural world on human communities, zoonotic transfer, agronomy, self‐sufficiency and food security
	Highlighting intersecting outcomes of conservation action and broader environmental priorities	As people reconsider the need and risks of international travel, local conservation programs and sites once again have special significance to local communities and should be promoted with these communities and broader environmental priorities (e.g. climate change) in mind	Local sites enable new experiences without the carbon footprint or public health risks that overseas travel will carry (CS2, CS3, CS5), although non‐domestic tourism‐dependent economies are likely to suffer if travel abates, meaning new financial models will need to be developed (e.g. CS1; CS6)
Cross‐sectoral engagement	Restructuring of local supply chains	Restructuring could provide an opportunity for communities to support a diversified range of more sustainable small‐scale activities, such as the selling and processing of food resources to local rather than international communities	In Sri Lanka (CS4), fish sellers were quick to adapt to the loss of traditional markets by, for example, shifting sales from central market locations to door to door, although consumer prices fluctuated, and were often much higher than usual. Restructuring could facilitate a more equitable spread of resources, by reducing the importance of a small number of traders who traditionally generate high profits, thus increasing resilience to future shocks
	Knowledge exchange	The emergence of COVID‐19 requires knowledge exchange across sectors to understand and quantify the risks posed to human and wildlife communities	In Cantanhez National Park (CS6), strict health protocols were communicated to park guards and implemented to enable them to continue biodiversity and health monitoring activities while minimising the risk of inter‐species transmission. Such training and best‐practice learnings can then be communicated to other conservation agencies, tour guides and local communities
Local partnerships	Creation of new partnerships	The COVID‐19 pandemic has illustrated the importance of collaboration between local and international partners, bi/multilateral partnerships and cooperation	In Seychelles, Guinea‐Bissau, Indonesia and other locations, the (temporary) absence/reduced presence of international researchers presents opportunities to enhance investment in training and education of local researchers, to strengthen their role in international projects and reduce reliance on international researchers (CS1, CS2, CS5, CS6)
Co‐design and delivery	Leveraging enhanced public interest in sustainability needs	Opportunities exist to leverage community action related to public concerns raised by the pandemic, including enhanced food and livelihood security, and sustainable living practices. The pandemic has been a powerful contextualiser and demonstrator for conservation messages regarding our interdependence on the natural world	In the United Kingdom, the pandemic has seen a rise in interest in areas of the Eden Project's expertise (CS3), such as horticulture and sustainable food production. The Cornwall Wildlife Trust (CS5) realised a 56% rise in website users during the UK lockdown period compared to the first quarter of 2020. These preliminary findings point to the potential for conservation organisations to expand their public reach
	Streamlining conservation for multiple benefits	Opportunities to address COVID‐19‐related problems may support a range of new actions with conservation benefits	Opportunities for health and well‐being in nature may emerge, such as the purchase of reserves located close to centres of population that are managed as much for people as wildlife (CS5). This in turn may take pressure off more biodiverse or sensitive sites. In Guinea‐Bissau (CS2) and Indonesia (CS6), the One Health approach will be used to communicate links between ecosystems, animal and human health with local communities, including risks of COVID‐19 to endangered great ape populations. The One Health concept also generates fundraising opportunities
Local investment and leadership	Local investment by regional and national governments	The COVID‐19 pandemic has highlighted the potential value of changes—as advocated by some communities—for implementing changes aimed at protecting against socio‐political shocks and enhancing livelihoods	In Sri Lanka (CS4), fishers say COVID‐19 has highlighted the paucity of adequate cold storage facilities, which reduces the quality of fish and profit margins. Financial assistance in areas of need identified by local communities could widen targeting and livelihood opportunities, potentially reducing incentives to target threatened species
	Restructuring of current systems of power and exploitation	Declines in food security, trade and tourism all risk a reduction in incentives to conserve local biodiversity. However, altered governance systems may present opportunities to prioritise conservation management in ways that benefit both biodiversity and communities, for example via the scaling up of traditional land‐management practices	At the Eden Project (CS3), the Emergence Academy is a forum for the creative, holistic and interdisciplinary exploration for permanent and wide‐scale solutions to the world's ‘wicked problems.’ Arguably the context for this has been laid bare by the current pandemic, which has renewed the appetite for, and belief in, the need and possibility of meaningful change as the world emerges from lockdown
Diverse income streams	Creation of sustainable tourism revenue	Authorities are likely to prioritise boosting of tourism revenues in the short term, which may have negative impacts upon biodiversity conservation and wider sustainability goals. At sites where reducing the numbers of visitors and interactive exhibits may be preferred over the long term, this could lead the way to offering packages that provide more sustained and meaningful knowledge exchange models	In tourism‐dependent economies such as the Seychelles (CS1), diversifying the tourism industry will require creative thinking and political support, for example, the outsourcing of tourism by using live feeds of biodiversity to international platforms or centres. At the Eden Project (CS3), reduced numbers of visits could pave the way for more bespoke packages in areas such as food production, horticulture, plant science, energy and sustainability and community engagement
	Diversification of income	More diversified and sustainable income flows at international, national and local scales are essential to enhance resilience in the face of sudden perturbation	At regional and local scales, the pandemic has demonstrated how quickly a source of funding can be cut‐off (e.g. face to face fundraising), and that diversified funding models are key (CS1, CS3, CS5, CS6)
Innovation	Novel research directions	The COVID‐19 pandemic has the potential to trigger further research into the impacts of tourism, human disturbance, wildlife disease and inter‐species transmission, human–wildlife interactions, self‐sufficiency and food security. Human responses to the pandemic also offer opportunities for greater understanding of the response and adaptations of human communities to shock, how resilience is affected and to what extent inherent flexibilities exist within social–ecological systems for responding to future perturbations	In the Seychelles (CS1), the temporary complete loss of international tourism presents unique opportunities for research into tourism impacts, development of new tourism models, diversification of tourism revenues and increasing the sustainability of the industry. In Sri Lanka (CS4), fisheries stakeholders explained that previous experience of perturbation events (e.g. civil war) facilitated their adaptation to COVID‐19, which could present learning points for other socio‐ecological systems. The One Health approach is particularly relevant in locations where substantial disease risks exist owing to sharing or landscapes (such as Guinea‐Bissau, CS2) and human encroachment on wild habitats, hunting and butchering of wild animals, trade in wild animals and animal parts, and the subsequent sale of meat in crowded markets with low hygiene levels. There is great potential for an increased focus upon inter‐species disease transmission and risk, including building on existing research to include COVID‐19 in project objectives (CS2, CS6)
	Inclusive conservation	New modes of interacting, catalysed by the pandemic, may initiate approaches that are more inclusive. Remote collaboration has the potential to engage a wider range of stakeholders, although in some rural areas infrastructure for remote networking is lacking	During lockdown, the Eden Project (CS3) explored the use of new digital platforms. This content recorded some of their highest online engagement figures (e.g. how‐tos in vegetable growing, ‘Kitchen Table’ conversations), and reached audiences not previously engaged with Eden's messaging. In Borneo, BNF initiated a series of webinars and developed online education sessions, some of which included pandemic‐related discussions (CS6)

## 4 DISCUSSION

Human‐induced and natural perturbations present ongoing challenges for meeting conservation needs. Our literature review and case studies suggest a number of similarities and important differences between past perturbations and the COVID‐19 pandemic. Understanding how the present pandemic will impact conservation in the short and long term, and to what extent these impacts differ from past perturbations, is vital to ensure the delivery of biodiversity conservation outcomes in an uncertain future.

### 4.1 Comparing conservation outcomes of past perturbations and COVID‐19

Economic losses, livelihood and food insecurity, and rapid changes to domestic and international trade are all features of past perturbations and the COVID‐19 pandemic (Santos, 2020; Sayer et al., 2012; Sunderlin et al., 2001; CS1‐CS6). During past perturbations, a sustained drop in tourism revenue or disruption of trade routes resulted in the decline in related industries and increased poverty. In some highly diverse regions, this led to an increase in resource use to unsustainable levels, accelerating the fragmentation of habitats with cascading impacts upon biodiversity conservation (Butsic et al., 2015; Dudley et al., 2002; Sayer et al., 2012). It is too early to understand the long‐ or even medium‐term consequences of declines in tourism and trade resulting from the COVID‐19 pandemic, although economic turmoil and cascading biodiversity impacts are likely and are already being observed in some regions (Corlett et al., 2020; Lindsey et al., 2020; CS1). In three of our case studies, the sudden cessation of tourism or visitor income to conservation sites and communities resulted in severe economic losses (CS1, CS3 and CS5; Bhammar et al., 2021; Lindsey et al., 2020). Similar to perturbations such as war, natural or nuclear disasters, the economic impact of COVID‐19 is likely to far outlast the day‐to‐day disruption of social distancing and mobility restrictions. Despite the development of multiple vaccines and the reduction in the rate of transmission that vaccination will bring, potential new virus variants, lingering (and legitimate) concerns about the safety of international travel, and the impact of austerity measures will impact spending power. Unless the pandemic can be effectively controlled at the global scale, isolation or distancing measures may need to be sporadically implemented and tourists or traders may stay away from sites due to ongoing restrictions or the fear of contracting or transmitting COVID‐19. Tourism operations may also need to be restricted to prevent inter‐species transmission of COVID‐19 to endangered wildlife. For the tourist industry in particular, this means future income streams may need to be restructured around fewer total visitors to a site at one time, while local communities and businesses will need to be prepared and supported in the case of sudden economic losses when local COVID‐19 cases rise (CS1, CS3 and CS5). While not yet observed in our case studies or the literature, it is possible that reduced visitation rates will necessitate higher ‘per visit’ charges to maintain income streams, with tourism to some locations ultimately becoming more exclusive and less accessible for the majority of people. While this might lead to reduced travel emissions from large volumes of tourists, it may potentially also lead to reduced opportunities to inspire large numbers of people about the natural world and its conservation. To maintain broad accessibility and public interest in such conservation sites, alternative methods of engagement—including adopting novel technologies and online opportunities—will thus need to be established.

Also common to past perturbations and the COVID‐19 pandemic is the difficulty of meeting research and conservation needs during large‐scale restrictions or upheaval (Conteh et al., 2017; Hilton et al., 2003, CS1, CS2, CS4 and CS6). A reduction in conservation monitoring and enforcement capacity is currently being observed as movements are restricted and funding postponed (Corlett et al., 2020; Evans et al., 2020; Gaynor et al., 2016; CS1, CS3 and CS5), whereas ongoing monitoring has been identified as critical for determining the impacts of the pandemic on local wildlife and people (Harrison et al., 2020; CS2, CS6). The resumption of funding and conservation programs to pre‐pandemic levels cannot be guaranteed, and may take many years to reach if international government and philanthropic donations are impacted by global financial downturns. As demonstrated by past perturbations and the COVID‐19 pandemic, the cessation of monitoring and research activities does not just impact upon research advances; it may also contribute to the loss of livelihoods, the loss of local infrastructure as repair work fails to be undertaken, the loss of skilled researchers to other professions and the erosion of hard‐won engagement and trust between conservation agencies and local communities, for which continued communication and a physical presence is vital (Conteh et al., 2017; Dudley et al., 2002; CS1, CS3, CS5 and CS6). The global‐scale cessation of human movement is, however, a unique feature of the present pandemic. While this situation has brought about novel research opportunities as well as challenges, it also places into perspective the current dependence of many conservation projects on the movement of researchers from higher income countries. Linking to wider debates and critiques of ‘parachute science’ (where international research is conducted with minimal local engagement or capacity building) and the recognised need to decolonise research and conservation, our case studies demonstrate the importance of investing in local partnerships and capacity building to maintain conservation activities in the face of perturbation. The same case studies also demonstrate the importance of data sharing and communication technologies to better facilitate international collaborations involving research in remote locations, particularly in developing countries (Eichhorn et al., 2020; CS1, CS2, CS4 and CS6).

During some past perturbations, monitoring and intervention activities occurred at very different spatial and temporal scales to the perturbation and its after‐effects, rendering them ineffective (Sayer et al., 2012). For example, past perturbations sometimes drove new conservation problems or monitoring needs that only become apparent months or years later, such as the transportation of radionuclides by wildlife after the Fukushima Daiichi disaster (Madigan et al., 2012). While it is still too early to understand if COVID‐19‐facing conservation interventions will be effective, it reinforces the importance of identifying recently emerged monitoring needs and conservation issues and acting upon these. This includes, for example, the potential for inter‐species transmission of the virus to wild great ape populations (CS2, CS6; Melin et al., 2020), the implications of intensive farming practices for rapid disease transmission and mutation rates, within and across species (i.e. mink farms, Levitt & Kevany, 2021), and the links between habitat destruction and zoonotic transmission (Carrington, 2020). Concerns remain that the COVID‐19 pandemic will provoke rushed government measures that harm conservation efforts, or create societal backlash towards species perceived to be a vector of zoonotic disease, with negative consequences for the local persistence of these species and their habitats (MacFarlane & Rocha, 2020). As the COVID‐19 pandemic is similar to—but of greater global public interest than—recent past zoonotic pandemics such as SARS, the present pandemic presents additional opportunities to reframe conservation monitoring as a public health benefit (Jones et al., 2008; Morse et al., 2012; Zinsstag et al., 2011) and to understand the biodiversity changes associated with the global scale of ‘human confinement’ and the long‐term conservation outcomes of pandemic‐related societal and behavioural changes (Bates et al., 2020; Cheval et al., 2020; Soga et al., 2021; CS1, CS2, CS3, CS5 and CS6).

### 4.2 Opportunities for biodiversity conservation in the wake of the COVID‐19 pandemic

The outcomes of COVID‐19 on biodiversity conservation are still emerging, but have the potential to be severe and wide‐ranging (Corlett et al., 2020; Lindsey et al., 2020; CS1–CS6). Despite this, the pandemic—and our responses—present opportunities to implement changes that will benefit conservation (Table 3). Our case studies highlighted opportunities for conservation in areas of research and knowledge acquisition (e.g. long‐term vision and innovation), management and policy (cross‐sectoral engagement, co‐design and co‐delivery) and education and community (local partnerships, local investment and leadership). These findings are also backed up by the emerging literature (Lindsey et al., 2020; Miller‐Rushing et al., 2021; Phua et al., 2021; Ramvilas et al., 2021; Roe et al., 2020; Smith et al., 2021).

Some opportunities will be relatively ‘easy’ gains, requiring little additional funding or societal change to implement. The creation of new partnerships, increased collaboration and cooperation between local and international partners and cross‐sector agencies, and enhanced use of technologies to facilitate data collection and sharing under restricted travel conditions are examples of relatively simple actions that have broad benefits for biodiversity conservation. Other opportunities, however, will require fundamental institutional change and extensive government and public engagement and support if they are to be realised. For example, the restructuring of local supply chains towards more diversified and sustainable activities, and the dismantling of current social‐political systems that exploit or disempower the most vulnerable human communities (Table 3).

### 4.3 Delivering biodiversity conservation in an uncertain future

The pandemic has highlighted the inherent vulnerabilities in the social and economic models upon which much conservation monitoring, research and tourism activities are based (Lindsey et al., 2020; Figure 2). In particular, communities across the world that were highly reliant upon international tourism saw the vast majority of their revenue and livelihoods halted almost overnight. For countries with functioning social welfare schemes in place, a sudden loss of business on this scale is a major economic blow that will take years to recover from (Deutsche Welle, 2020, CS1, CS3 and CS5); but for places without such societal safety nets, or where the COVID‐19 pandemic has added to existing societal catastrophes, the cumulative impacts are quickly turning into escalating food security concerns and major human health crises (Karasapan, 2020; The World Bank, 2020, CS2 and CS4). It is important not to minimise the loss and pain that the current pandemic has caused, and will continue to cause, for so many. Yet, one aspect that has been repeatedly raised in the recent popular and scientific literature on COVID‐19 is the interconnectedness of all things—of our reliance upon healthy, intact and functioning ecosystems; the risks of intensive exploitation of the natural world; and our vulnerability in the face of ecosystem and biodiversity loss (Carrington, 2020; Grandcolas & Justine, 2020; Harrison et al., 2020; Vidal, 2020). This suggests that those who are able to rebuild, or are in a position to help others rebuild, be they governments, conservation organisations, research institutions or local communities, should use the pandemic to reconsider the status quo for biodiversity conservation (Table 3; Figure 2) and as a ‘learnable moment for conservation’ (Schwartz et al., 2020). The pandemic prompts us to reflect upon what changes are needed to better protect and restore global biodiversity and how this can be achieved in socially responsible and equitable ways. In so doing, we have the opportunity to move forward with a renewed emphasis upon effective management and research practices, enhancing (capacity for) local leadership of research projects, and promoting behaviours and actions that are resilient to future perturbations.

## 5 CONCLUSION

COVID‐19 is not the first, nor the last pandemic that our global society will have to deal with (Hymas et al., 2021), yet in many ways we are living through a unique situation in recent global history. COVID‐19 has underlined the increasing fragility of our world and global society as we continue to exploit nature for short‐term economic gains. Many of the emerging and anticipated outcomes for biodiversity conservation arising from the COVID‐19 pandemic look bleak, but as the literature and our case studies demonstrate, myriad opportunities also exist. All ecosystems, whether or not they are protected, are facing the climate crisis, the biodiversity loss crisis and other global environmental problems. The COVID‐19 pandemic has been the only recent threat that has made the whole world pause; in this pause, we must take stock, and do our best to provide people with the information, ideas and capacity to safeguard our natural heritage for a post‐pandemic future.

## CONFLICT OF INTEREST

The authors declare no competing interests.

## AUTHORS' CONTRIBUTIONS

R.H.T., K.J.H., J.S.U.H., R.E., C.N.K.‐B., F.v.V. and N.B. conceptualised the study; R.H.T., K.J.H., J.S.U.H., E.B., C.C., D.D., F.F.‐D., Y.E., G.G., M.E.H., M.A.I., D.R.K., C.N.K.‐B., C.M., M.‐M.M., A.N., F.v.V., I.W. and N.B. involved in investigation; R.H.T., K.J.H., J.S.U.H., R.E., C.N.K.‐B. and N.B. led the writing of the original draft; R.H.T., K.J.H., J.S.U.H., E.B., C.C., R.E., D.D., F.F.‐D., Y.E., G.G., M.E.H., M.A.I., D.R.K., C.N.K.‐B., C.M., M.‐M.M., A.N., A.R.d.B., F.v.V., I.W. and N.B. involved in reviewing and editing of the manuscript.

## Supporting information

Supplementary Material

Supplementary Material

## Data Availability

This manuscript does not use any primary data.
