# Supplementary material for: Envisioning a resilient future for biodiversity conservation in the wake of the COVID‐19 pandemic
Source: People Nat (Hoboken). 2021 Sep 28;3(5):990–1013. doi: 10.1002/pan3.10262 (PMC8661774; doi:10.1002/pan3.10262)
Supplement: Supplementary file 1 — Supplementary Material [file PAN3-3-990-s002.docx]

**Supplementary Materials 1: Literature extracted from Web of Knowledge searches.**

**Natural disasters:**

Affan M, Fadli N, Jufriadi J, Nazaruddin N, Sofyan H, Nizamuddin N, Marzuki M, SaphaI D (2019) IOP Conference Series: Earth and Environmental Science 348, 012108.

Hilton GM, Atkinson PW, Gray GAL, Arendt WJ, Gibbons DW (2003) Rapid decline of the volcanically threatened Montserrat oriole. Biological Conservation 111, 79–89.

Hirayama H, Tomita M, Hara K (2020) Quantitative monitoring of changes in forest habitat connectivity following the great eastern Japan earthquake and tsunami. Landscape Ecology 35, 1519–1530.

Katoh K, Yoshikawa T, Kamijo T, Higuchi H (2020) Relationship between vegetation structure and avian communities on Miyakejima Island, Japan, 13 years after a major volcanic eruption. Pacific Science 74, 1–18.

Kurosawa T (2021) Facility against tsunamis and green infrastructure—a case study of post-disaster reconstruction after the Great East Japan Earthquake. Coastal Engineering Journal, DOI: 10.1080/21664250.2021.1877916.

Main MA, Dearden P (2007) Tsunami impacts on Phuket's diving industry: geographical implications for marine conservation. Coastal Management 35, 467-481.

Marske KA, Ivie MA, Hilton GM (2007) Effects of volcanic ash on the forest canopy insects

of Montserrat, West Indies. Environmental Entomology 36, 817-825.

Maxwell SL, Butt N, Maron M, McAlpine CA, Chapman S, Ullmann A, Segan DB, Watson JEM (2019) Conservation implications of ecological responses to extreme weather and climate events. Diversity and Distributions 25, 613–625.

Porwal MC, Padalia H, Roy PS (2012) Impact of tsunami on the forest and biodiversity richness in Nicobar Islands (Andaman and Nicobar Islands), India. Biodiversity Conservation 21, 1267–1287.

Reynolds MH, Berkowitz P, Klavitter JL, Courtot KN (2017) Lessons from the Tōhoku tsunami: A model for island avifauna conservation prioritization. Ecology and Evolution 7, 5873–5890.

Viña A, Chen X, McConnell WJ, Liu W, Xu W, Ouyang Z, Zhang H, Liu Z (2011) Effects of natural disasters on conservation policies: the case of the 2008 Wenchuan Earthquake, China. AMBIO 40, 274–284.

Zhang Z, Yuan S, Qi D, Zhang M (2014) The Lushan earthquake and the giant panda: impacts and conservation. Integrative Zoology 9, 376–378.

**Technical perturbations:**

Deryabina TG, Kuchmel SV, Nagorskaya LL, Hinton TG, Beasley JC, Lerebours A, Smith JT (2015) Long-term census data reveal abundant wildlife populations at Chernobyl. Current Biology 25, R811–R826.

Irving GJ, Round PD, Savini T, Lynam AJ, Gale GA (2018) Collapse of a tropical forest bird assemblage surrounding a hydroelectric reservoir. Global Ecology and Conservation 16, e00472.

Møller AP, Mousseau TA (2007a) Species richness and abundance of birds in relation to radiation at Chernobyl. Biology Letters 3, 483-486.

Møller AP, Mousseau TA (2007b) Determinants of interspecific variation in population declines of birds from exposure to radiation at Chernobyl. Journal of Applied Ecology 44, 909-919.

Møller AP, Mousseau TA (2011) Conservation consequences of Chernobyl and other nuclear accidents. Biological Conservation 144, 2787-2798.

Yoshida Y, Lee HS, Trung BH, Tran DH, Lall MK, Kakar K, Xuan TD (2020) Impacts of mainstream hydropower dams on fisheries and agriculture in Lower Mekong Basin. Sustainability 12, 2408.

**War or violent conflict:**

Beare D, Hölker F, Engelhard GH, McKenzie E, Reid DG (2010) An unintended experiment in fisheries science: a marine area protected by war results in Mexican waves in fish numbers-at-age. Naturwissenschaften 97, 797–808.

Brito JC, Durant SM, Pettorelli N, Newby J, Canney S, Algadafi W, Rabeil T, et al (2018) Armed conflicts and wildlife decline: Challenges and recommendations for effective conservation policy in the Sahara-Sahel. Conservation Letters 11, e12446.

Butsic V, Baumann M, Shortland A, Walker S, Kuemmerle T (2015) Conservation and conflict in the Democratic Republic of Congo: the impacts of warfare, mining, and protected areas on deforestation. Biological Conservation 191, 266-273.

Calle-Rendon BR, Moreno F, Hilário RR (2018) Vulnerability of mammals to land-use changes in Colombia’s post-conflict era. Nature Conservation 29, 79–92.

Canavire-Bacarreza G, Diaz-Gutierrez JE, Hanauer MM (2018) Unintended consequences of conservation: Estimating the impact of protected areas on violence in Colombia. Journal of Environmental Economics and Management 89, 46–70.

Castro-Nuñez A, Mertz O, Buritica A; Sosa CC, Lee ST (2017) Land related grievances shape tropical forest-cover in areas affected by armed-conflict. Applied Geography 85, 39-50.

Clerici N, Armenteras D, Kareiva P, Botero R, Ramírez-Delgado JP, Forero-Medina G, Ochoa J, Pedraza C, Schneider L, Lora C, Gómez C, Linares M, Hirashiki C, Biggs D (2020) Deforestation in Colombian protected areas increased during post-conflict periods. Scientific Reports 10, 4971.

Coates P (2014) Borderland, no-man's land, nature's wonderland: troubled humanity and untroubled earth. Environment and History 20, 499-516.

Conteh A, Gavin MC, McCarter J (2017) Assessing the impacts of war on perceived conservation capacity and threats to biodiversity. Biodiversity Conservation 26, 983-996.

Dávalos LM (2001) The San Lucas mountain range in Colombia: how much conservation is owed to the violence? Biodiversity and Conservation 10, 69–78.

De Merode E, Smith KH, Homewood K, Pettifor R, Rowcliffe M, Cowlishaw G (2007) The impact of armed conflict on protected-area efficacy in Central Africa. Biology Letters 3, 299–301.

Draulans D, Van Krunkelsven E (2002) The impact of war on forest areas in the Democratic Republic of Congo. Oryx 36, 35-40.

Dudley JP, Ginsberg JR, Plumptre AJ, Hart JA, Campos LC (2002) Effects of war and civil strife on wildlife and wildlife habitats. Conservation Biology 16, 319-329.

Dutta A (2020) Forest becomes frontline: conservation and counter-insurgency in a space

of violent conflict in Assam, Northeast India. Political Geography 77, 102117.

Enaruvbe GO, Keculah KM, Atedhor GO, Osewole AO (2019) Armed conflict and mining induced land-use transition in northern Nimba County, Liberia. Global Ecology and Conservation 17, e00597.

Gaynor KM, Fiorella KJ, Gregory GH, Kurz DJ, Seto KL, Withey LS, Brashares JS (2016) War and wildlife: linking armed conflict to conservation. Frontiers in Ecology and the Environment 14, 533-542.

Gorricho J, Schultze-Kraft M (2021) Wartime protected area governance: the case of Colombia’s Alto Fragua Indiwasi National Park. Third World Quarterly, DOI: 10.1080/01436597.2021.1892482.

Goswami R, Ganesh T (2014) Carnivore and herbivore densities in the immediate aftermath of ethno-political conflict: The case of Manas National Park, India. Tropical Conservation Science 7, 475-487.

Grima N, Singh SJ (2019) How the end of armed conflicts influence forest cover and subsequently ecosystem services provision? An analysis of four case studies in biodiversity hotspots. Land Use Policy 81, 267-275.

Hanson T (2018) Biodiversity conservation and armed conflict: a warfare ecology perspective. Annals of the New York Academy of Sciences 1429, 50-65.

Hanson T, Brooks TM, Da Fonseca GAB, Hoffmann M, Lamoreux JF, Machlis G, Mittermeier CG, Mittermeier RA, Pilgrim JD (2009) Warfare in biodiversity hotspots. Conservation Biology 23, 578-587.

Hart T, Hart J, Fimbel C, Fimbel R, Laurance WF, Oren C, Struhsaker TT, Rosenbaum HC, Walsh PD, Razafindrakoto Y, Vely M, DeSalle R (1997) Conservation and civil strife: two perspectives from Central Africa. Conservation Biology 11, 308-314.

Lawrence M, Stemberger H, Zolderdo A, Struthers D, Cooke S (2015) The effects of modern war and military activities on biodiversity and the environment. Environmental Reviews 23, 443-460.

Lindenmayer DB, MacGregor C, Wood J, Westgate MJ, Ikin K, Foster C, Ford F, Zentelis R (2016) Bombs, fire and biodiversity: Vertebrate fauna occurrence in areas subject to military training. Biological Conservation 204, 276–283.

Lindsell JA, Klop E, Siaka AM (2011) The impact of civil war on forest wildlife in West Africa: mammals in Gola Forest, Sierra Leone. Oryx 45, 69-77.

Loucks C, Mascia MB, Maxwell A, Huy K, Duong K, Chea N, Long B, Cox N, Seng T (2009) Wildlife decline in Cambodia, 1953–2005: exploring the legacy of armed conflict. Conservation Letters 2, 82-92.

Mazaris MD (2017) Manifestation of maritime piracy as an additional challenge for global conservation. Marine Policy 77, 171-175.

McNeely JA (2003) Conserving forest biodiversity in times of violent conflict. Oryx 37, 142-152.

Nguyen TT (2009) Vietnam and the environment: problems and solutions. International Journal of Environmental Studies 66, 1–8.

Richardson CJ, Hussain NA (2006) Restoring the Garden of Eden: an ecological assessment of the marshes of Iraq. Bioscience 56, 477-489.

Stevens K, Campbell L, Urquhart G, Dramer D, Qi J (2011) Examining complexities of forest cover change during armed conflict on Nicaragua’s Atlantic Coast. Biodiversity Conservation 20, 2597–2613.

Suarez A, Árias-Arévalo PA, Martínez-Mera E (2017) Environmental sustainability in post-conflict countries: insights for rural Colombia. Environment, Development and Sustainability 20, 997–1015.

Yong Sung C, Park C-B, Kim J-S (2019) Politics of forest fragmentation: a multiscale analysis on the change in the structure of forest landscape in the North and South Korean border region. Regional Environmental Change 19, 137–147.

Zúñiga-Upegui P, Arnaiz-Schmitz C, Herrero-Jáuregui C, Smart SM, López-Santiago CA, Schmitz MF (2019) Exploring social-ecological systems in the transition from war to peace: a scenario-based approach to forecasting the post-conflict landscape in a Colombian region. Science of the Total Environment 695, 133874.

**Sudden economic or political change:**

Bragina EV, Ives AR, Pidgeon AM, Kuemmerle T, Baskin LM, Gubar YP, Piquer-Rodriquez M, Keuler NS, Petrosyan BG, Radeloff VC (2015) Rapid declines of large mammal populations after the collapse of the Soviet Union. Conservation Biology 29, 844-853.

Hostert P, Kuemmerle T, Prishchepov A, Sieber A, Lambin EF, Radeloff VC (2011) Rapid land use change after socio-economic disturbances: the collapse of the Soviet Union versus Chernobyl. Environmental Research Letters 6, 045201.

Mikulić K, Radović A, Kati V, Jelaska SD Tepić N (2014) Effects of land abandonment on bird communities of smallholder farming landscapes in post-war Croatia: implications for conservation policies. Community Ecology 15, 169-179.

Montana M, Mlambo D (2019) Environmental awareness and biodiversity conservation among resettled communal farmers in Gwayi Valley Conservation Area, Zimbabwe. International Journal of Sustainable Development & World Ecology 26, 242–250.

Newton AC (2011) Social-ecological resilience and biodiversity conservation in a 900-year-old protected area. Ecology and Society 16(4):13.

Sayer JA, Endamana D, Ruiz-Perez M, Boedhihartono AK, Nzooh Z, Eyebe A, Awono A, Usongo L (2012) Global financial crisis impacts forest conservation in Cameroon. The International Forestry Review 14, 90-98.

Sunderlin WD, Angelsen A, Resosudarmo DP, Dermawan A (2001) Economic crisis, small farmer well-being, and forest cover change in Indonesia. World Development 29, 767-782.

Trappe J, Kunz F, Weking S, Kamp J (2017) Grassland butterfly communities of the Western Siberian forest steppe in the light of post-Soviet land abandonment. Journal of Insect Conservation 21, 813–826.

**Disease outbreaks:**

Arora S, Bhaukhandi KD, Mishra PK (2020) Coronavirus lockdown helped the environment to bounce back. Science of the Total Environment 742, 140573.

Baudron F, Liegéios F (2020) Fixing our global agricultural system to prevent the next COVID-19. Outlook on Agriculture 49, 111–118.

Bhammar H, Li W, Molina CMM, Hickey V, Pendry J, Narain U (2021) Framework for sustainable recovery of tourism in protected areas. Sustainability 13, 2798.

Bicca-Marques JC, Santos de Freitas D (2010) The role of monkeys, mosquitoes, and humans in the occurrence of a yellow fever outbreak in a fragmented landscape in South Brazil: protecting howler monkeys is a matter of public health. Tropical Conservation Science 3, 78-89.

Booth H, Arias M, Brittain S, Challender DWS, Khanyari M, Kuiper T, Li Y, Olmedo A, Oyanedel R, Pienkowski T, Milner-Gulland EJ (2021) “Saving lives, protecting livelihoods, and safeguarding nature”: risk-based wildlife trade policy for sustainable development outcomes post-COVID-19. Frontiers in Ecology and Evolution 9, 639216.

Borzée A, McNeely J, Magellan K, Miller JRB, Porter L, Dutta T, Kadinjappalli KP, Sharma S, Shahabuddin G, Aprilinayati F, Ryan GE, Hughes A et al. (2020) COVID-19 highlights the need for more effective wildlife trade legislation. Trends in Ecology & Evolution 35, 1052-1055.

Calistri P, Decaro N, Lorusso A (2021) SARS-CoV-2 pandemic: not the first, not the last. Microorganisms 9, 433.

Campos FS, Lourenço-de-Moraes R (2020) Ecological fever: the evolutionary

history of Coronavirus in human-wildlife relationships. Frontiers in Ecology and Evolution 8, 575286.

Cherkaoui S, Boukherouk M, Lakhal T, Aghzar A, El Youssfi L (2020) Conservation amid COVID-19 pandemic: ecotourism collapse threatens communities and wildlife in Morocco. E3S Web of Conferences 183, 01003.

Cheval S, Adamescu CM, Georgiadis T, Herrnegger M, Piticar A, Legates DR (2020) Observed and potential impacts of the COVID-19 pandemic on the environment. International Journal of Environmental Research and Public Health 17, 4140.

Cooke S, Twardek WM, Lynch AJ, Cowx IG, Oldn JD, Funge-Smith S, Lorenzen K, Arlinghaus R, Chen Y, Weyl OLF, Nyboer EA, Pompeu PS, et al (2021) A global perspective on the influence of the COVID-19 pandemic on freshwater fish biodiversity. Biological Conservation 253, 108932.

Corlett RT, Primack RB, Devictor V, Maas B, Goswami VR, Bates AE, Pin Koh L, Regan TJ, Loyola R, Pakeman RJ, Cumming GS, Pidgeon A, Johns D, Roth R (2020) Impacts of the coronavirus pandemic on biodiversity conservation. Biological Conservation 246: 108571.

Cumming T, Seidl A, Emerton L, Spenceley A, Kroner RG, Uwineza Y, van Zyl H (2021) building sustainable finance for resilient protected and conserved areas: lessons from COVID-19. Parks 27, 149-160.

Cunningham AA, Daszak P, Wood JLN (2017) One Health, emerging infectious diseases and wildlife: two decades of progress? Philosophical Transactions of the Royal Society: Biological Sciences 372, 20160167.

D'Cruze N, Green J, Elwin A, Schmidt-Burbach J (2020) Trading tactics: time to rethink the global trade in wildlife. Animals 10, 2456.

De Vos A, Cumming GS, Cumming DHM, Ament JM, Baum J, Clements HS, Grewar JD, Mciejewski K, Moore C (2016) Pathogens, disease, and the social-ecological resilience of protected areas. Ecology and Society 21(1): 20.

Evans KL, Ewen JG, Guillera-Arroita G, Johnson JA, Penteriani V, Ryan SJ, Sollmann R, Gordon IJ (2020) Conservation in the maelstrom of Covid-19 – a call to action to solve the challenges, exploit opportunities and prepare for the next pandemic. Animal Conservation 23, 235-238.

Gbogbo F, Kyei MO (2017) Knowledge, perceptions and attitude of a community living around a colony of straw-coloured fruit bats (*Eidolon helvum*) in Ghana after Ebola virus disease outbreak in West Africa. Zoonoses Public Health 64, 628–635.

Harrison ME, Wijedasa LS, Cole LES, Cheyne SM, Choiruzzad SAB, Chua L, Dargie GC, Ewango CEN, Honorio Coronado EN, Ifo SA, Imron MA, Kopansky D, Lestarisa T, O’Reilly PJ, et al (2020) Tropical peatlands and their conservation are important in the context of COVID-19 and potential future (zoonotic) disease pandemics. PeerJ 8: e10283.

Huang Q, Wang F, Yang H, Valitutto M, Songer M (2021) Will the COVID-19 outbreak be a turning point for China’s wildlife protection: New developments and challenges of wildlife conservation in China. Biological Conservation 254, 108937.

Hymas O, Rocha B, Guerrero N, Torres M, Ndong K, Walters G (2020) There’s nothing new under the sun – Lessons conservationists could learn from previous pandemics. Parks 27, 25-40.

Jones N, McGinlay J, Jones A, Malesios C, Holtvoeth J, Dimitrakopoulos PG, Gkoumas V,

Kontoleon A (2021) COVID-19 and protected areas: impacts, conflicts, and possible management solutions. Conservation Letters, e12800.

Kavousi J, Goudarzi F, Izadi M, Gardner CJ (2020) Conservation needs to evolve to survive in the post-pandemic world. Global Change Biology, In press.

Kishimoto K, Kobori H (2021) COVID-19 pandemic drives changes in participation in citizen science project “City Nature Challenge” in Tokyo. Biological Conservation 255, 109001.

Laffoley D, Baxter JM, Amon DJ, Claudet J, Hall-Spencer JM, Grorud-Colvert K, Levin LA, Reid PC, Rogers AD, Taylor ML, Woodall LC, Andersen NF (2020) Evolving the narrative for protecting a rapidly changing ocean, post-COVID-19. Aquatic Conservation: Marine and Freshwater Ecosystems 1, 1–23.

Lindsey P, Allan J, Brehony P, Dickman A, Robson A, Begg C, et al (2020) Conserving Africa’s wildlife and wildlands through the COVID-19 crisis and beyond. Nature Ecology and Evolution 4, 1300-1310.

Lu M, Wang X, Ye H, Wang H, Qiu S, Zhang H, Liu Y, Luo J, Feng J (2021) Does public fear that bats spread COVID-19 jeopardize bat conservation? Biological Conservation 254, 108952.

MacFarland D, Rocha R (2020) Guidelines for communicating about bats to prevent persecution in the time of COVID-19. Biological Conservation 248, 108650.

Manenti R, Mori E, Di Canio V, Mercurio S, Picone M, Caffi M, Brambilla M, Ficetola GF, Rubolini D (2020) The good, the bad and the ugly of COVID-19 lockdown effects on wildlife conservation: Insights from the first European locked down country. Biological Conservation 249, 108728.

McGinlay J, Gkoumas V, Holtvoeth J, Fuertes RFA, Bazhenova E, Benzoni A, Botsch K, Martel CC, CC, Cervera I, Chaminade G, Doerstel J, et al (2020) The impact of COVID-19 on the management of European protected areas and policy implications. Forests 11, 1214.

Miller-Rushing AJ, Athearn N, Blackford T, Brigham C, Cohen L, Cole-Will R, Edgar T, Ellwood ER, Fisichelli N, Pritz CF, Gallinat AS, Gibson A, Hubbard A, McLane S, Nydick K, Primack RB, Sachs S, Super PE (2021) COVID-19 pandemic impacts on conservation research, management, and public engagement in US national parks. Biological Conservation 257, 109038.

Novelli M, Gussing Burgess L, Jones A, Ritchie BW (2018) ‘No Ebola…still doomed’ – The Ebola-induced tourism crisis. Annals of Tourism Research 70, 76-87.

Olival KJ, Epstein JH, Wang L, Field HE, Daszak P (2012) Are bats unique viral reservoirs? In: New Directions in Conservation Medicine: Applied Cases of Ecological Health. Aguirre AA, Ostfeld RS, Daszak P (Editors). Oxford University Press, pp. 195-212.

Phua C, Andradi-Brown DA, Mangubhai S, Ahmadia GN, Mahajan SL, Larsen K, Friel S, Reichelt R, Hockings M, Gill D, et al (2021) Marine protected and conserved areas in the time of COVID. Parks 27, 85-102.

Pinder AC, Raghavan R, Britton JR, Cooke SJ (2020) COVID-19 and biodiversity: the paradox of cleaner rivers and elevated extinction risk to iconic fish species. Aquatic Conservation: Marine and Freshwater Ecosystems 30, 1061–1062.

Ramvilas G, Dhyani S, Kumar B, Sinha N, Raghavan R, Selvara G,Divakar N, Anoop VK, Shalu K, Sinha A, Kulkarni A, Das S, Molur S (2021) Insights on COVID-19 impacts, challenges and opportunities for India’s biodiversity research: from complexity to building adaptations. Biological Conservation 255, 109003.

Roe D, Dickman A, Kock R, Milner-Gulland EJ, Rihoy E, Sas-Rolfes M’t (2020) Beyond banning wildlife trade: COVID-19, conservation and development. World Development 136, 105121.

Schwartz MW, Gilkman JA, Cook CN (2020) The COVID-19 pandemic: A learnable moment for conservation. Conservation Science and Practice 2, e255.

Singh K, Singh RP, Tewari SK (2020) Ecosystem restoration: challenges and opportunities for India. Restoration Ecology 29, e13341.

Smith MKS, Smit IPJ, Swemmer LK, Mokhatla MM, Freitag S, Roux DJ, Dziba L (2021) Sustainability of protected areas: vulnerabilities and opportunities as revealed by COVID-19 in a national park management agency. Biological Conservation 255, 108985.

Soga M, Evans MJ, Cox DTC, Gaston KJ (2021) Impacts of the COVID-19 pandemic on human–nature interactions: pathways, evidence and implications. People and Nature, https://doi.org/10.1002/pan3.10201.

Sugai LSM (2020) Pandemics and the need for automated systems for biodiversity monitoring. The Journal of Wildlife Management 84, 1424–1426.

Terraube J, Fernández-Llamazares Á (2021) Strengthening protected areas to halt biodiversity loss and mitigate pandemic risks. Current Opinion in Environmental Sustainability 46, 35–38.

Turcios-Casco MA, Cazzolla Gatti R (2020) Do not blame bats and pangolins! Global consequences for wildlife conservation after the SARS-CoV-2 pandemic. Biodiversity and Conservation 29, 3829–3833.

Waithaka J, Dudley N, Álvarez M, Mora SA, Chapman S, Figgis P, Fitzsimons J, Gallon S, Gray TNE, Kim M, Pasha MKS, Perkin S, Roig-Boixeda P, Sierra C, Valverde A, Wong M (2021) Impacts of COVID-19 on protected and conserved areas: A global overview and regional perspectives. Parks 27, 41-56.

Wood CL, McInturff A, Young HS, Kim D, Lafferty KD (2017) Human infectious disease burdens decrease with urbanization but not with biodiversity. Philosophical Transactions of the Royal Society: Biological Sciences 372, 20160122.

Wu T, Perrings C (2017) Conservation, development and the management of infectious disease: Avian influenza in China, 2004–2012. Philosophical Transactions of the Royal Society B: Biological Sciences 372, 2004-2012.

Wu T, Perrings C, Shang C, Collins JP, Daszak P, Kinzig A, Minteer BA (2020) Protection of wetlands as a strategy for reducing the spread of avian influenza from migratory waterfowl. Ambio 49, 939–949.

Wu T (2021) The socioeconomic and environmental drivers of the COVID-19 pandemic: a review. Ambio 50, 822–833.

Yang H, Ma M, Thompson JR, Flower RJ (2020) Protect the giant ibis through the pandemic. Science 369, 929.

**Supplementary Materials 2: Case study locations and impacts of COVID-19 on biodiversity conservation outcomes.**

*Case study 1 (CS1): Seychelles*

Methodology: The impacts described in Tables 1 and 2 were drawn from reported direct experiences of the observed and expected impacts of COVID-19 by government officials and staff of the Public Trust managing the most visited natural site in the country. These are supported by publicly available national data and reports (e.g., National Statistics Bureau, Seychelles Central Bank). The information is considered accurate, albeit incomplete. The knowledge gaps are mainly the lag period between data gathering and publication, e.g., at the time of writing, information is not yet available for 2021.

Overview: The Seychelles is an archipelago of over 100 islands in the Western Indian Ocean. The country is heavily dependent on global tourism (with 384,204 tourism arrivals in 2019 compared to a population of 97,625; Seychelles National Bureau of Statistics 2019) and has high levels of biodiversity, endemism, and threatened species. Almost half (47.6%; Seychelles MEECC Geo-database, 2020) of the country’s land area is protected, amongst the highest in the world. Rapidly implemented anti-COVID-19 measures in March 2020 included the cancellation of all international passenger flights, the closure of Seychelles airport to passenger traffic, and no foreign nationals permitted to enter the country. Tourism dropped to zero in late March 2020, and public movement restrictions were implemented for just over 3 weeks. As an island state, with full control over its borders, it was possible to eliminate the virus from the country in the early stages of the pandemic. Since this first partial lockdown and tourism restrictions, the country has made significant progress in fully vaccinating 61% of the total population (Ministry of Health Seychelles 2021) and has cautiously re-opened its borders to tourists. Despite these measures, the Seychelles experienced a major COVID-19 outbreak in the second quarter of 2021 when confirmed cases surged to the highest case rates in the world.

Political decisions and societal behaviours to prevent COVID-19 transmission entail few positive and substantial negative impacts for biodiversity conservation in the Seychelles, with impacts likely to vary in their duration (Tables 1 and 2). Examples of impacts include: temporary total cessation and medium-term reduction in tourism income to conservation and the national economy, temporary declines in wildlife disturbance from people and traffic, increased national biosecurity risks due to greater pressure to import perishable goods by air, discontinuation of key biodiversity monitoring, re-scheduled fund disbursement and cancellation of funded conservation projects (but also new small-scale funding opportunities), decline in international research work, and compromised partnerships (Table 1).

*Case study 2: Cantanhez National Park, Guinea-Bissau*

Methodology: The impacts described in Tables 1 and 2 were identified based upon UNEP data gathered on Guinea-Bissau cashew market exports, including prices, and the reported direct experiences of local informants living or working within Cantanhez National Park (CNP) with whom we discussed the impact of COVID-19 on their lives and daily activities. Key stakeholders at IBAP (Instituto da Biodiversidade e das Áreas Protegidas) who manage research and conservation activities at CNP were also consulted on the observed and expected impacts of COVID-19, as well as the non-government organisation the National Association for Local Development (NADEL), which runs health campaigns, including for COVID-19, within CNP. Given the range of sources and close connections of the above stakeholders with CNP during the COVID-19 pandemic, the derived information is considered accurate.

Overview: Guinea-Bissau, West Africa, is among the world’s most economically vulnerable countries. Cantanhez National Park (CNP), Guinea-Bissau, is located in the Tombali region in the south of the country bordering the Republic of Guinea-Conakry. The park is 1067 km^2^ and is inhabited by approximately 24,000 people, most of whom rely on the income generated from cashew farming, a major driver of deforestation (African Development Bank 2018; Havik et al. 2018). It is the country’s most biodiverse protected area, with numerous threatened species persisting within forest fragments surrounded by human settlements. CNP is divided into different zones including core areas, buffer zones and sustainable development zones. Legal and illegal hunting occur inside CNP. However, due to tolerance and local protective beliefs some species such as the Critically Endangered western chimpanzee (*Pan troglodytes verus*) are not hunted. The chimpanzee population in central CNP attract some wildlife tourism, although revenue is limited and local communities cannot rely on tourism profits for livelihoods.

To halt the spread of COVID-19, authorities in Guinea-Bissau implemented various measures including the closure of land borders, a ban on international flights and national travel restrictions, limitations on the size of gatherings, and the use of masks in public spaces. Research and tourism in CNP was halted, although local park guards were permitted to continue biodiversity and health monitoring activities, with a strict health protocol followed. Political and economic decisions to prevent COVID-19 transmission have impacted conservation in a number of ways (Tables 1 and 2). These include: training and changes in behaviour of park rangers due to increased concern and focus upon inter-species disease transmission and risk, cessation of social science data collection due to COVID-19 restrictions, and food insecurities due to loss of international markets, leading to an increased reliance upon natural resources. Cessation of international travel for research has renewed emphasis on the importance of in-country collaboration, and national expertise for research capacity.

*Case study 3: Eden Project Limited, Cornwall, UK*

Methodology: The below information and content in Tables 1 and 2 was gathered directly from data held by the Eden Project, and through discussion of observed and expected impacts of the COVID-19 pandemic with Eden’s science and engagement teams. Given the direct nature of the sources, the derived information (observed and expected) is considered to be highly accurate and reliable.

Overview: The Eden Project is an educational charity which aims to connect people with the living world and each other. Since opening in 2001, the Eden Project has welcomed over 22 million visitors to its 55-acre headquarter site in the southwest of the UK, which consists of gardens and indoor tropical and Mediterranean ‘biomes’. Eden has put more than two billion GBP into the local economy, and maintains a number of conservation projects and sites around the world. In 2019 the Eden Project directly employed a staff of 450 full-time equivalent, with a turnover of 28 million GBP, making it one of the largest private employers in Cornwall. In line with national lock-down measures, Cornwall’s Eden Project site was closed for 75 days, with estimated revenue losses of 4.5–5 million GBP. Just four horticulturalists, an emergency executive and a handful of health and safety personnel remained actively working across the site. Eden re-opened on 6 June 2020, with a maximum daily visitor intake of 1250, a third of average equivalent days in summer. Eden’s site in Cornwall is supported financially through admissions, catering, retail and event spend and the site does not need on-going public subsidy to support its operations. Any funding the team secure via Charitable Trusts and grants will support additional activity over and above the core destination. This includes Eden’s major community outreach project – The Big Lunch. This work was able to continue during the lockdown period.

National and international political decisions and societal behaviours to prevent COVID-19 transmission have highlighted substantial challenges for the Eden Project in Cornwall, its wider projects and for tourism in Cornwall and the UK, some of which are likely to continue across months or even years (Tables 1 and 2). These primarily include the cessation of tourist income, skeleton staffing at its sites, and the redirection of current and future public funds. More positive impacts include the temporary lack of disturbance to wildlife and the opportunity to increase attention to its charitable mission and messaging (Table 1). Whilst the impact of covid on the Eden operation is the short-term was difficult, the pandemic did allow Eden to assess its operating model. Innovations such as operating a timed ticketed model to manage social distancing will remain in place and will support a more efficient operating model for the site that offers certainty on how many visits and when visitors are coming.

*Case study 4: Sri Lanka’s offshore fishing fleet*

Methodology: Semi-structured interviews (n=19) were conducted at two landing sites for multi-day vessels in the south west coast of Sri Lanka during June 2020. Interviews were conducted in the local language (Sinhalese) and sampling was purposive to target individuals engaged in catching, trading and retail of shark products. Interviewees were often already known to the interviewer (co-author IW), who has worked within each landing site since January 2019. Questions were designed to explore 1) changes in volume and economics of trade during the COVID-19 lockdown, 2) changes to management and governance, and 3) impact of the changes on livelihoods. All questions were open-ended and the interviewer included additional prompts and questions to allow for deeper exploration of issues. All interviewees were informed that data collection was anonymous and would be kept confidential. A verbal consent to participate was provided to enable those with lower levels of literacy to participate, and interviews were conducted under an ethics permit from the University of Exeter (eCORN001727 v6.1). Study limitations, including the number of interviews and choice of sites being located within fairly close proximity (~100km) of each other, limit the representativeness of findings for all of Sri Lanka. Furthermore, the short timeframe of data collection (1 month) and shifting social situation due to COVID-19 means results should be interpreted in context.

Overview: Fishing is the primary livelihood for many in Sri Lanka and provides the most important animal protein source for the majority (~67%) of the population (Herath et al. 2019). The national fishing fleet predominantly comprises small vessels operating in coastal areas, however, substantial landings originate from the offshore fleet locally referred to as multi-day vessels. These multi-day vessels primarily target large pelagics such as skipjack tuna (*Katsuwonus pelamis*), but also regularly land shark species of conservation concern including silky (*Carcharhinus falciformus*) and mako (*Isurus spp.*), and their crew have been arrested with large shark catches in other country’s waters (Martin et al. 2013; Collins et al. 2021). In response, prohibitions on landing of thresher (Alopiidae spp.) and oceanic white-tip (*C. longimanus*), and mandatory log-book reporting and satellite-tracking systems have been introduced over the last decade (Herath et al. 2019; Collins et al. 2020).

Landings of sharks are sold primarily via auction at landing sites for both domestic consumption (predominantly consumed dried), and export. Measures introduced on 20 March 2020 to control the spread of COVID-19 included restricting access to landing sites on a permit basis, which prevented consumers and some small-scale traders from entering (Azmy et al. 2021). All restrictions were lifted from 28 June 2020 onwards however, the impact on fishing communities on the south west coasts was exacerbated by localised clusters of COVID-19 infections which resulted in sporadic regional lockdown restrictions. This exploratory study identified the following potential impacts of COVID-19: unequal but significant livelihood impacts, a disparity in responses and resilience across supply chains, rise in consumer prices, and potential reduction in monitoring of illegal activities e.g., landing of prohibited species, and ongoing fishery data collection issues (Tables 1 and 2). More positively, a decline in international trade in wildlife products of conservation concern was also observed.

*Case study 5: The Cornwall Wildlife Trust, UK*

Methodology: The below information and content in Tables 1 and 2 was gathered directly from data held by the Cornwall Wildlife Trust (CWT), and through discussion of observed and expected impacts of the COVID-19 pandemic with CWT’s employees and volunteers. Given the direct nature of the sources, the derived information is considered accurate and reliable.

Overview: Cornwall Wildlife Trust (CWT) is an independent charity whose aim is to protect the county of Cornwall's wildlife and wild places. The strategic aims of the CWT are to conserve habitats, to protect, record and monitor wildlife, and to inspire and engage members of the public through raising awareness of Cornwall’s wildlife. The CWT has 17,000 members, employs 77 full time equivalent staff and relies on the input of a network of hundreds of regular active volunteers. The CWT works in partnerships with stakeholders and delivers advice to support conservation and enhance sustainable practices across Cornwall. The CWT also works to collate, manage and disseminate biological and geological information for use in research, conservation and sustainable development. The CWT manages 57 terrestrial Nature Reserves across Cornwall covering >5500 acres, and are involved as partners or leads on projects that work with stakeholders to protect rare or declining species, both terrestrial and marine. The Nature Reserves act as core areas to protect and enhance a diverse range of wildlife including both common and rare species. CWT are also involved as partners or leads on the Cornwall Beaver Project, which recently reintroduced this extirpated species to the South-West of England, a badger vaccination against bovine TB programme, which works with farmers in mid-Cornwall, and a county-wide hedgehog conservation initiative.

The COVID-19 pandemic has fundamentally impacted on the way CWT operates and seeks to pursue its charitable objectives. These include, a significant loss of income and staff capacity, the need to restructure office work and travel, temporary closure of nature reserves, the cancellation of community and fundraising events, and the need to re-focus priorities and develop a recovery plan (Tables 1 and 2).

*Case study 6: Forest and Peatland Conservation, Central Kalimantan, Indonesia*

Methodology: The impacts described in Tables 1 and 2 were identified based upon the reported direct experiences of the BNF Indonesia management and field teams, including through their normal dialogues with multiple external stakeholders and partners, ranging variously from government officials to schools, local village community and conservation team members, plus interpretation of government and other (e.g., IUCN SSC PSG SGA 2021) guidelines and local media reports. The impressions derived through this are considered highly accurate in terms of impacts on BNF and its activities, and are expected to be generally reliable in terms of indirect impacts, given the range of sources from which this information is derived and the perceived lack of motivation for any verbal/informal sources to provide untruthful information.

Overview: Borneo is part of the Sundaland biodiversity hotspot (Myers et al. 2000). It has a particularly rich flora and fauna, including 37 endemic bird and 44 endemic mammal species (MacKinnon et al. 1996), and a total of 825 terrestrial plant and 198 terrestrial animal species classified as threatened by the IUCN. Most of the island lies in Indonesian territory, split into five provinces. Central Kalimantan is the largest province by area and is the location of the Borneo Nature Foundation’s (BNF) research and conservation activities. Its wildlife includes the critically endangered Bornean orangutan (*Pongo pygmaeus*), for which the largest proportion of the remaining population is found in Central Kalimantan (Wich et al. 2008). Despite this conservation importance, Kalimantan has experienced an estimated 144,000 km^2^ of forest loss between 1973 and 2015 (Gaveau et al. 2016), due to agricultural conversion, particularly for oil palm and pulp wood, timber harvesting, and mining, in addition to the drainage and increased fire risk in peatland areas (Miettinen et al. 2012; Gaveau et al. 2016; Miettinen et al. 2016; Dohong et al. 2017). This presents a complex array of challenges for conservationists in the region (Harrison et al. 2020a). The Indonesian government’s response to the COVID-19 pandemic has involved declaring a public health emergency on 31^st^ March 2020; restricting international and domestic travel and international research permits; implementing a series of large-scale social restrictions, including temporary closures of schools and work places, restrictions on religious activities and non-essential business in some regions, to be implemented by regional governments based on approval from the Minister of Health (Widodo 2020); plus implementing, facemask-wearing and financial stimulus policies as part of a National Economic Recovery programme representing ~3.8% of national GDP, including increased benefits for low-income households, tax reliefs and reductions, and capital injections into state-owned enterprises (IMF 2021); and recently (January 2021) initiating a nationwide vaccination programme.

As in tropical peatland areas across the globe (Harrison et al. 2020b), the COVID-19 pandemic has impacted the research, conservation, community development and outreach activities of the BNF in myriad ways, with impacts felt from early on in the pandemic (BNF 2020), which have evolved over time as the pandemic has continued and evolved locally, and as teams and work programmes adapt to the “new normal”. Examples from BNF’s work in and around the peat-swamp forest of Sebangau National Park illustrate the effects of the pandemic for this conservation NGO and the local community (Tables 1 and 2). These include: ongoing impacts on research efforts and general work activities; initial temporary suspension of and subsequent ongoing restrictions to in-person children’s education and other group activities, including workshops, events and community development training and socialisation initiatives; economic impacts within the local community, potential short- to medium term benefits and risks to local habitats and wildlife associated with changes in human activity, and opportunities to highlight the importance of environmental conservation for public health. While some of these impacts have been possible to mitigate to some extent (e.g. through delivering education packs and online sessions to local children who would have joined group sessions pre-pandemic: Hutasoit 2021), effectiveness may be reduced.

**References:**

African Development Bank (2018) Country Gender Profile: Guinea-Bissau. Available at: <https://www.afdb.org/fileadmin/uploads/afdb/Documents/Generic-Documents/Guinea-Bissau_-_Country_gender_profile.pdf>. Last accessed 1 July 2020.

Azmy N, Giritharan A, Jamel H, Mangubhai S, de Vos A (2021) The impacts of COVID-19 lockdowns on coastal fisheries in Sri Lanka. Oceanswell, Colombo. DOI: 10.13140/RG.2.2.12909.10725/1.

BNF (2020) Statement by Borneo Nature Foundation concerning the CoVID-19 virus pandemic. Borneo Nature Foundation. Available at: <http://www.borneonaturefoundation.org/en/news/statement-by-borneo-nature-foundation-concerning-the-covid-19-virus-pandemic/>. Last accessed 26 March 2020.

Collins C, Bech Letessier T, Broderick A, Wijesundara I, Nuno A (2020) Using perceptions to examine human responses to blanket bans: The case of the thresher shark landing-ban in Sri Lanka. Marine Policy, 104198.

Collins C, Nuno A, Broderick AC, Curnick DJ, de Vos A, Franklin T, et al. (2021) Understanding persistent non-compliance in a remote, large-scale marine protected area. Frontiers in Marine Science 8, 503.

Dohong A, Aziz AA, Dargusch P (2017) A review of the drivers of tropical peatland degradation in South-East Asia. Land Use Policy 69, 349-360.

Gaveau DLA, Sheil D, Husnayaen, Salim MA, Arjasakusuma S, Ancrenaz M, Pacheco P, Meijaard E (2016) Rapid conversions and avoided deforestation: examining four decades of industrial plantation expansion in Borneo. Scientific Reports 6, 32017.

Harrison ME, Ottay JB, D’Arcy LJ, Cheyne SM, Anggodo, Belcher C, Cole L, Dohong A, Ermiasi Y, Feldpausch T, Gallego-Sala A, Gunawan A, Höing A, Husson SJ, Kulu IP, Soebagio SM, Mang S, Mercado L, Morrogh-Bernard HC, Page SE, Priyanto R, Ripoll Capilla B, Rowland L, Santos E, Schreer V, Sudyana IN, Bin Bakeri Taman S, Thornton SA, Upton C, Wich SA, van Veen F (2020a) Tropical forest and peatland conservation in Indonesia: Challenges and directions. People and Nature 2, 4-28.

Harrison ME, Wijedasa LS, Cole LES, Cheyne SM, Choiruzzad SAB, Chua L, Dargie GC, Ewango CEN, Honorio Coronado EN, Ifo SA, Imron MA, Kopansky D, Lestarisa T, O’Reilly PJ, Offelen JV, Refisch J, Roucoux K, Sugardjito J, Thornton SA, Upton C, Page S (2020b) Tropical peatlands and their conservation are important in the context of COVID-19 and potential future (zoonotic) disease pandemics. PeerJ 8: e10283.

Havik PJ, Monteiro F, Catarino S, Correia AM, Catarino L, Romeiras MM (2018) Agro-economic transitions in Guinea-Bissau (West Africa): historical trends and current insights. Sustainability 10(10), 3408.

Herath HLN, Hewapathirana HP, Gunawardane ND, Friedman K (2019) Understanding food security, livelihoods in a changing shark and ray fisheries sector in Sri Lanka, Fisheries and Aquaculture Circular. Rome, 1185.

Hutasoit PA (2021) Environmental education strategies during the pandemic. Borneo Nature Foundation.

IMF (2021) Policy Responses to COVID-19: Indonesia (as of 5 May 2021). International Monetary Fund. Retrieved 16th May 2021.

IUCN SSC PSG SGA (2021) Great Apes, COVID-19 and the SARS CoV-2: April 2021 revision, IUCN Species Survival Commission Primate Specialist Group, Section on Great Apes.

MacKinnon K, Hatta G, Halim H, Mangalik A (1996) The Ecology of Kalimantan, Indonesian Borneo. Singapore: Periplus Editions (HK) Ltd.

Martin S, Moir Clark J, Pearce J, Mees C (2013) Catch and bycatch composition of illegal fishing in the British Indian Ocean Territory (BIOT). Available at: <http://www.iotc.org/documents/update-catch-and-bycatch-composition-illegal-fishing-british-indian-ocean-territory-ukot>.

Miettinen J, Hooijer A, Shi C, Tollenaar D, Vernimmen R, Liew SC, Malins C, Page SE (2012) Extent of industrial plantations on Southeast Asian peatlands in 2010 with analysis of historical expansion and future projections. Global Change Biology Bioenergy 4, 908-918.

Miettinen J, Shi C, Liew SC (2016) Land cover distribution in the peatlands of Peninsular Malaysia, Sumatra and Borneo in 2015 with changes since 1990. Global Ecology and Conservation 6, 67–78.

Ministry of Health Seychelles (2021) Available at: <http://www.health.gov.sc/wp-content/uploads/COVID-19-VACCINATION-UPTAKE-REPORT_5TH-MAY-2021.pdf>. Last accessed 19 May 2021.

Myers N, Mittermeier RA, Mittermeier CG, da Fonseca GAB, Kent J (2000) Biodiversity hotspots for conservation priorities. Nature 403, 853-858.

Wich SA, Meijaard E, Marshall AJ, Husson S, Ancrenaz M, Lacy RC, van Schaik CP, Sugardjito J, Simorangkir T, Traylor-Holzer K, Doughty M, Supriatna J, Dennis R, Gumal M, Knott CD, Singleton I (2008) Distribution and conservation status of the orangutan (Pongo spp.) on Borneo and Sumatra: how many remain? Oryx 42, 329-339.

Widodo J (2020) Peraturan Presiden Republik Indonesia Nomor 21 Tahun 2020 tentang Pembatasan Sosial Berskala Besar Dalam Rangka Percepatan Penanganan Corona Virus Disease 2019 (COVID-19) [Regulation of the President of Indonesia No. 21 Year 2020 regarding Large-Scale Social Restrictions in the Context of Accelerating the Handling of Corona Virus Disease 2019 (COVID-19)]. Jakarta, Indonesia, President of the Republic of Indonesia.
