# Supplementary material for: Envisioning a resilient future for biodiversity conservation in the wake of the COVID‐19 pandemic
Source: People Nat (Hoboken). 2021 Sep 28;3(5):990–1013. doi: 10.1002/pan3.10262 (PMC8661774; doi:10.1002/pan3.10262)
Supplement: Supplementary file 2 — Supplementary Material [file PAN3-3-990-s001.pdf]

PEOPLE AND NATURE

How will COVID-19 impact biodiversity conservation?

Ruth H. Thurstan, Kimberley J. Hockings, Johanna S.U. Hedlund, Elena Bersacola, Claire Collins, Regan Early, Yunsiska Ermiasi, Frauke Fleischer-Dogley, Gabriella Gilkes, Mark E. Harrison, Muhammad Ali Imron, Christopher N. Kaiser-Bunbury, Daniel Refly Katoppo, Cheryl Marriott, Marie-May Muzungaile, Ana Nuno, Aissa Regalla de Barros, Frank van Veen, Isuru Wijesundara, Didier Dogley & Nancy Bunbury.

The COVID-19 pandemic has had tremendous repercussions for our global economy and society, and these impacts will continue to resonate over the coming years and decades. Less explored are the outcomes of the COVID-19 pandemic for global biodiversity conservation efforts, and how observed impacts of the pandemic on conservation activities compare to previous societal, economic, technical and natural perturbations.

In an attempt to plug this knowledge gap, our study uses the published literature and six case studies from around the world to identify the emerging and anticipated outcomes of COVID-19 for biodiversity conservation efforts. We compare these to observed conservation outcomes of different types of perturbations affecting a range of scales, locations and economies, including natural disasters, nuclear accidents, violent conflicts and sudden socioeconomic shifts.

We find similarities, but also key differences, between the current pandemic and other types of perturbation. The emerging literature on the impacts of COVID-19 and our case studies highlight the inherent vulnerabilities that exist in the ways in which we conduct conservation. Most immediate include the issues of maintaining funding sources and conservation capacity throughout the pandemic and during the rebuilding phase. Longer-term, the loss of expertise and continuous monitoring data may reduce the ability of conservationists and local stakeholders to fully engage with or contribute to decision-making processes.

COVID-19 is not the first perturbation to bring significant social and economic disruption with impacts on wildlife, habitats, incomes and livelihoods, as well as conservation practice, management, research and funding. Yet it may be the first perturbation to bring the whole world to a temporary standstill, and we may yet experience profound and long-term behavioural and cultural shifts resulting from the pandemic. Such shifts will necessitate changes in the way we manage and fund conservation of our wildlife and wild spaces, and conservationists will need to rise to the challenges the pandemic brings. We can do this by identifying best practice from prior perturbations, and by identifying and acting upon the opportunities that the COVID-19 global pandemic presents. Enhanced collaborations and partnerships at local levels, cross-sectoral engagement, local investment and leadership will all enhance the resilience of conservation efforts in the face of future perturbations. Other resilience-inducing actions are also possible, but will require fundamental institutional change and extensive engagement and support if they are to be realised.

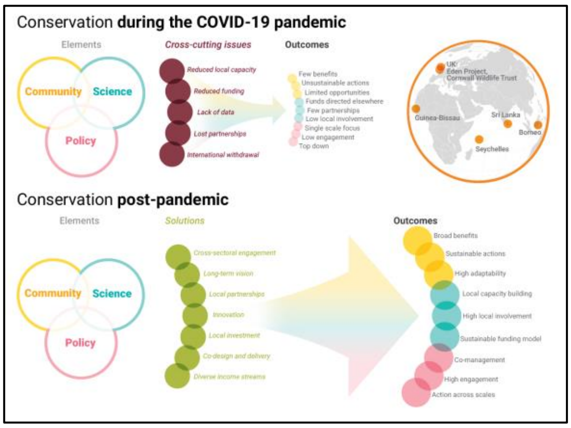

Can we change the course of biodiversity conservation post-pandemic? Adapted from Figure 2 in the published paper. With thanks to Nigel Hawtin for producing the illustration.

The COVID-19 pandemic has not yet run its course, and the direct and indirect impacts of the disease will undoubtedly continue to present major stumbling blocks to biodiversity conservation efforts. However, by attempting to learn from the current pandemic and past perturbations, we have the opportunity to reconsider the status quo for conservation. In so doing, perhaps we can promote behaviours and actions that are resilient to future perturbations and which maximise the positive outcomes for biodiversity conservation and ourselves.
